# Synergy of Iron Chelators and Therapeutic Peptide Sequences Delivered via a Magnetic Nanocarrier

**DOI:** 10.3390/jfb8030023

**Published:** 2017-06-26

**Authors:** Gayani S. Abayaweera, Hongwang Wang, Tej B. Shrestha, Jing Yu, Kyle Angle, Prem Thapa, Aruni P. Malalasekera, Leila Maurmann, Deryl L. Troyer, Stefan H. Bossmann

**Affiliations:** 1 Department of Chemistry, Kansas State University, Manhattan, KS 66041, USA; gsa2014a@gmail.com (G.S.A.); hongwang@ksu.edu (H.W.); yujing0415@ksu.edu (J.Y.); kja2665@truman.edu (K.A.); aruni@ksu.edu (A.P.M.); leila76@ksu.edu (L.M.); 2 Department of Anatomy & Physiology, Kansas State University, Manhattan, KS 66041, USA; tbs3@ksu.edu (T.B.S.); troyer@vet.k-state.edu (D.L.T.); 3 Microscopy and Analytical Imaging Laboratory, University of Kansas, Lawrence, KS 66045, USA; psthapa@ku.edu

**Keywords:** iron/iron oxide core/shell nanoparticle, breast cancer, therapeutic peptide sequence, iron chelator, cell viability study

## Abstract

Here, we report the synthesis, characterization, and efficacy study of Fe/Fe_3_O_4_-nanoparticles that were co-labeled with a tumor-homing and membrane-disrupting oligopeptide and the iron-chelator Dp44mT, which belongs to the group of the thiosemicarbazones. Dp44mT and the peptide sequence PLFAERL(_D_[KLAKLAKKLAKLAK])CGKRK were tethered to the surface of Fe/Fe_3_O_4_ core/shell nanoparticles by utilizing dopamine-anchors. The 26-mer contains two important sequences, which are the tumor targeting peptide CGKRK, and _D_[KLAKLAK]_2_, known to disrupt the mitochondrial cell walls and to initiate programmed cell death (apoptosis). It is noteworthy that Fe/Fe_3_O_4_ nanoparticles can also be used for MRI imaging purposes in live mammals. In a first step of this endeavor, the efficacy of this nanoplatform has been tested on the highly metastatic 4T1 breast cancer cell line. At the optimal ratio of PLFAER_D_[KLAKLAK]_2_CGKRK to Dp44mT of 1 to 3.2 at the surface of the dopamine-coated Fe/Fe_3_O_4_-nanocarrier, the IC_50_ value after 24 h of incubation was found to be 2.2 times lower for murine breast cancer cells (4T1) than for a murine fibroblast cell line used as control. Based on these encouraging results, the reported approach has the potential of leading to a new generation of nanoplatforms for cancer treatment with considerably enhanced selectivity towards tumor cells.

## 1. Introduction

Cancer has become one of the leading causes of death worldwide. The major cause of cancer death in women is breast cancer [1]. In a woman’s lifetime there is a 1 in 7 in risk of developing breast cancer [2]. Even though there are several treatment methods for breast cancer, such as hormone therapy, aromatase inhibitors and cytotoxins, there is still improvement needed in specifically targeting cancer cells to reduce side effects and toxicity in normal cells [2]. For successful cancer treatment, it is essential to deliver chemotherapeutic agents in effective doses to tumor cells minimizing adverse effects to normal healthy cells [3]. Cancer cells have different growth characteristics than normal cells. To supply nutrients for the excessive growth of the cells, leaky blood vessels are formed, due to the (partial) absence of pericytes lining the endothelial cells. These blood vessels have pore sizes of 100–600 nm, which can be advantageous, allowing nanoparticles to diffuse into tumor cells (Enhanced Permeation and Retention [4]). Therefore, chemotherapeutic agents coupled with nanoparticles can be, principally, used as successful drug carriers into tumor cells, increasing drug tumor accumulation [3]. However, serious doubts have been cast recently on the efficacy of the EPR effects in humans [5]. Therefore, peptide targeting sequences or other moieties should be employed to utilize active transport processes for drug delivery [4,5].

Blood vessels express specific endothelial markers, which are not prominent in normal cells [6]. Ligands such as peptides can bind to these vascular markers and when these ligands are bound to drugs they can be concentrated in tumor cells, increasing efficacy and reducing adverse effects to normal cells [3,6]. Inspired by Ellerby et al. [7], we have combined two peptide sequences, CGKRK, which targets blood vessels in tumors, but not vessels of normal tissues [8], and _D_[KLAKLAK]_2_ [6], which is a pro-apototic sequence that disrupts membranes, to create a chimeric peptide [6,7]. _D_[KLAKLAK]_2_ is capable of disrupting mitochondrial membranes, thus leading to apoptosis of cancer cells [6]. We have chosen a derivative of the iron-chelator Dp44mT [9,10,11,12,13] as a small molecule drug, because it is known to target several pathways in tumors. PLFAERL is cleaved by calpains, Ca^2+^-dependent cytosolic proteases that are overexpressed in virtually all solid tumors.[14] Calpain-mediated cleavage will release the therapeutic peptide sequence AERL_D_[KLAKLAK]_2_ CGKRK from the Fe/Fe_3_O_4_-nanocarrier upon entering the cytoplasm.

By conjugating PLFAERL _D_[KLAKLAK]_2_ CGKRK to the surface of an iron/iron oxide (Fe/Fe_3_O_4_) nanoparticle [15,16,17,18,19] we can actively target tumors. Iron is an important element for multiple cellular processes, as for instance proliferation, and DNA synthesis [20,21]. Dp44mT (Di-2-pyridylketone-4, 4, -dimethyl-3-thiosemicarbazone) is known to effectively chelate iron(II) and iron(III), generate reactive radicals[10], and interact with several pathways: (1) Dp44mT regulates the AMPK-dependent energy homeostasis pathway in cancer cells [11]; (2) Dp44mT induces endoplasmic reticulum (ER) stress and activates PERK/eIF3α, IRE1α, ATF6, and calmodulin kinase, resulting in ER-stress-associated pro-apoptotic signaling [12]; (3) Dp44mT decreases the efficacy of mitochondrial peroxidases, such as peroxiredoxin-3 (Prx3), which are important for cell survival in multiple tumors; (4) Dp44mT can selectively inhibit topoisomerase II**α** in (breast) cancer cells, leading to G_1_ cell cycle arrest at nanomolar concentrations [9]. The dopamine-tethered Dp44mT-derivative is released from the surface of Fe/Fe_3_O_4_ nanocarriers, because the dopamine-anchors are replaced by glutathione and other thiol-containing peptides and proteins in the cytoplasm.

The underlying paradigm of this research is that by combining CGKRK, _D_[KLAKLAK] and a Dp44mT-derivative and tethering the resulting therapeutic payload to a dopamine-coated Fe/Fe_3_O_4_ nanocarrier, we will be able to target the tumor vasculature of breast tumors and then the mitochondria in breast cancer cells. We will then activate several signaling cascades triggering apoptosis: The _D_[KLAKLAK]_2_ sequence will disrupt the mitochondrial cell walls [6]. Dp44mT will regulate the AMPK-dependent pathway [11], create ER-stress [12], reduce mitochondrial peroxidase activity and cause G_1_ cell cycle arrest [13]. AERL_D_[KLAKLAK]_2_ CGKRK and the Dp44mT-derivative will be released from the nanocarrier after dopamine-coated Fe_3_O_4_-nanoparticles have escaped from early endosomes into the cytoplasm, as previously demonstrated [15].

As a first step of designing an optimal targeting system for treating breast cancer, the activity of the nanocarrier was tested on the highly metastatic 4T1 murine breast cancer cell line. The results were compared with a non-cancerous murine skin fibroblast cell line (MSFs). The therapeutic payload was successfully tethered to the nanoparticle by means of a stable amide bonds with the dopamine ligand, which binds to the Fe_3_O_4_ shell of the core/shell nanoparticles with very high affinity [17,18,22]. The major objective of the comparative cell culture experiments was to determine the best loading ratio of PLFAERL _D_[KLAKLAK]_2_ CGKRK to the Dp44mT-derivative at the Fe/Fe_3_O_4_-nanoparticle surface. Furthermore, we have demonstrated the suitability of the Fe/Fe_3_O_4_-nanoparticles as MRI imaging agents in BALB/c mice bearing 4T1 tumors.

## 2. Results

### 2.1. Characterization of PLFAERL _D_[KLAKLAK]_2_CGKRK by HPLC Purification and MALDI-TOF

The purity of PLFAERL _D_[KLAKLAK]_2_CGKRK was determined by HPLC to 94.6 percent, based on its UV-absorption at 200 nm. It is generally accepted that peptides of purity >90% can be used without further precipitation in cell experiments. The corresponding HPLC chromatogram is reported in the SI section (Figure S1). The MALDI-TOF (matrix-assisted laser desorption time-of-flight) mass spectrum shows peaks at 2921.77, 2922.77, 2923.77, 2924.77 g·mol^−1^. The calculated molecular weight of the 26-mer PLFAERL _D_[KLAKLAK]_2_CGKRK is 2923.87 g·mol^−1^. The most abundant peak 2922.77 g·mol^−1^ is due to the loss of a hydrogen atom. The second most abundant peak is the molecular ion peak. The mass spectrum can be found in the SI section (Figure S2).

### 2.2. Analysis of the Linking Efficacy of Dopamine-Glutaric Acid-Modified PLFAERL _D_[KLAKLAK]_2_CGKRK and the Dp44mT-Derivative to Fe/Fe_3_O_4_-nanoparticles via ICP

The iron and sulfur content of the Fe/Fe_3_O_4_-nanocarriers was determined by means of an inductively coupled plasma (ICP) torch with emission detection. The results are summarized in Table 1. The iron content of the dopamine-coated Fe/Fe_3_O_4_-nanocarriers was in the range from 71 to 72.8%, which is typical, based on comparisons with earlier findings [17]. The sulfur content of dopamine-covered Fe/Fe_3_O_4_-nanoparticles was virtually zero. Therefore, the amount of added dopamine-glutaric acid-modified PLFAER_D_[KLAKLAK]_2_**C**GKRK and the Dp44mT-derivative could be determined by means of an ICP standard procedure, after a stepwise addition procedure. This is possible, because both, therapeutic peptide sequence (cysteine) and Dp44mT-derivative contain (after addition to dopamine) one sulfur molecule each.

**Scheme 1:**
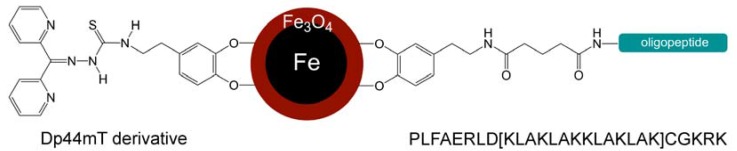
Dopamine-coated Fe/Fe_3_O_4_-nanocarriers carrying both a therapeutic peptide sequence and a Dp44mT-derivative (thiosemicarbazone).

**Figure 1:**
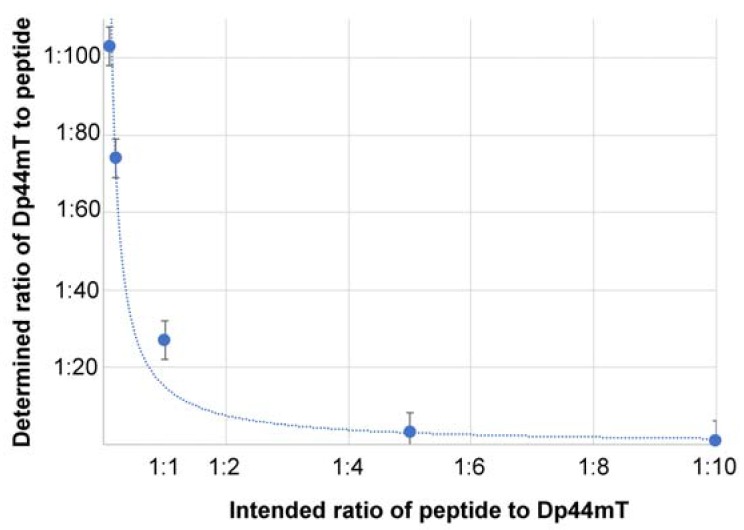
Intended molar ratio of PLFAER_D_[KLAKLAK]_2_CGKRK to Dp44mT-derivative at the surface of the Fe/Fe_3_O_4_-nanocarriers vs. the experimentally determined ratio. The maximal error is shown for each measurement. The curve represents the best data fit.

It is noteworthy that the efficacy of the addition of dopamine-glutaric acid-modified PLFAER_D_[KLAKLAK]_2_**C**GKRK to oleylamine/octadecene-covered Fe/Fe_3_O_4_ is significantly lower than of the reaction of compound **3** with Fe/Fe_3_O_4_-surface bound dopamine (see Scheme 1), most likely because of competition between dopamine and the dopamine-anchored peptide sequence in step 2 of the attachment procedure. In contrast, there is no competing reaction in the addition of compound **3** to dopamine. As shown in Figure 1, the efficacy of this reaction increases with increased concentration of compound **3**, as anticipated for a chemical potential-driven reaction.

### 2.3. Transmission Electron Microscopy of the Fe/Fe_3_O_4_-Nanocarriers

**Figure 2:**
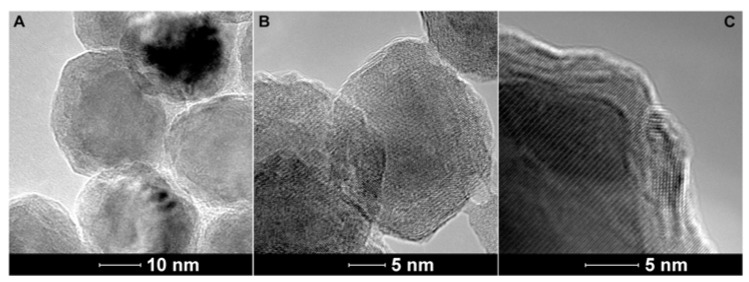
TEM of dopamine-stabilized Fe/Fe_3_O_4_-nanoparticles. (**A**): Dop-Fe/Fe_3_O_4_ Peptide/Dp44mT (10:1); (**B**) and (**C**): Dop-Fe/Fe_3_O_4_ Peptide/Dp44mT (1:5) (see Table 1).

**Figure 3:**
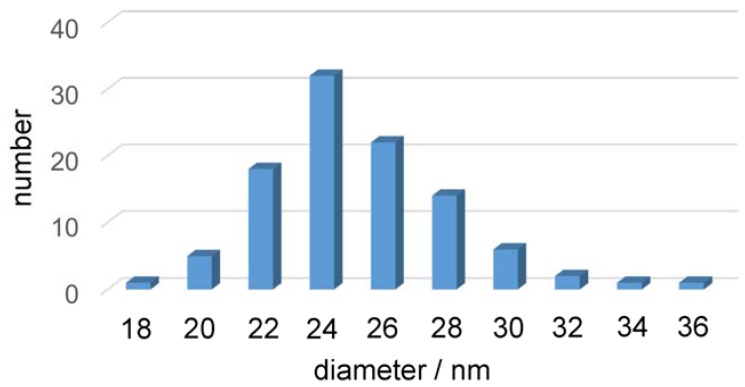
Size-distribution of Dop-Fe/Fe_3_O_4_ Peptide/Dp44mT (1:5) (see Table 1) according to TEM imaging results.

The Fe/Fe_3_O_4_-nanocarriers were characterized by TEM. As shown in Figure 2, the core/shell structure of the nanoparticles is clearly discernible. The inner Fe(0) cores are crystalline, albeit not monocrystalline. They consist of several crystalline domains. A histogram of the size distribution of the Fe/Fe_3_O_4_-nanocarriers is shown in Figure 3.

From Figure 3 it can be discerned that the average size of the Fe/Fe_3_O_4_-nanocarriers is 24 ± 4 nm (standard deviation). The Fe_3_O_4_ shells are in average 1.5 nm thick. This is consistent with an average diameter of the Fe(0) cores of 21 nm.

### 2.4. Dynamic Light Scattering (DLS) and Zeta-Potential Determination of the Fe/Fe_3_O_4_-Nanocarriers

As Table 2 indicates, all Fe/Fe_3_O_4_-nanocarriers feature similar hydrodynamic diameters, as determined by DLS in phosphate-buffered saline (PBS). This is an indication for clustering of the dopamine-coated nanoparticles. Previous studies have indicated that this clustering is concentration dependent and, principally, reversible [17]. The original data can be found in the SI section (Figures S3 to S9). The Zeta-potential is the electrokinetic potential of a nanoparticle at its slipping plane. For nanoparticles, higher zeta potentials will result in more stability and reduced aggregation. Therefore, particles with highly negative or highly positive zeta potentials (>±40–60 mV) are often longterm-stable. In contrast, nanoparticles with lower zeta potentials can readily aggregate and have, therefore, only moderate stability (<±0–40 mV) [9,10]. In agreement with our expectations, the primary amine functions of the dopamine-covered Fe/Fe_3_O_4_-nanoparticles cause positive zeta-potentials, albeit only in the moderate range. This finding can be regarded as a mechanistic explanation why clustering is observed by means of DLS. The original zeta potential measurements are summarized in the SI Section (Figures S10 to S16).

### 2.5. Cell Viability Assays

**Figure 4:**
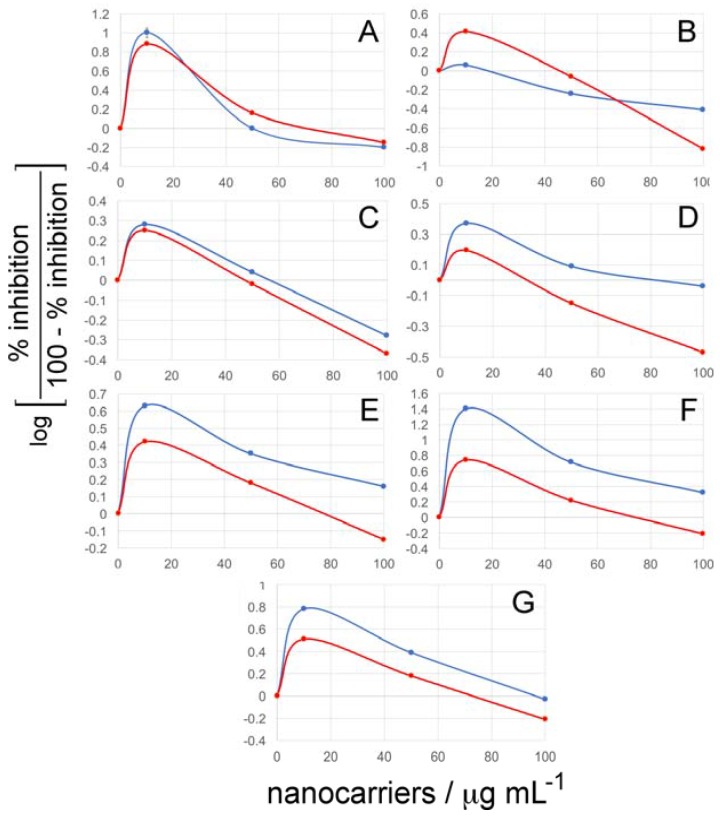
Growth inhibition of 4T1 cells (red) and murine fibroblasts (blue) after 24 h of incubation as a function of nanocarrier concentration. (**A**): dopamine-coated Fe/Fe_3_O_4_; (**B**): dopamine-coated Fe/Fe_3_O_4_-Peptide; All other graphs: dopamine-coated Fe/Fe_3_O_4_-Peptide/Dp44mT; (**C**): Peptide/Dp44mT: 1/1.1; (**D**): 1/3.2; (**E**): 1/27; (**F**): 1/74; (**G**): 1/103. The experimental errors in all curves are negligible (<±3 percent).

The toxicity of the Fe/Fe_3_O_4_-nanocarriers against the murine breast cancer cell line 4T1 and a normal murine fibroblast cell line was tested as described in the Methods Section. The results are summarized in Figure 4.

It is noteworthy that for five nanocarriers, the growth inhibition of 4T1 breast cancer cells is significantly greater than for non-cancerous murine fibroblast cells, whereas dopamine-coated Fe/Fe_3_O_4_ is equally toxic to both cell types and dopamine-coated Fe/Fe_3_O_4_-Peptide nanocarriers are less toxic to 4T1 cells at lower concentrations and more toxic at higher concentrations. Our results clearly indicate that the absolute and relative concentrations of dopamine-glutaric acid-modified PLFAER_D_[KLAKLAK]_2_**C**GKRK and dopamine-attached Dp44mT derivative on the surface of Fe/Fe_3_O_4_ play an important role with respect to targeting cancer cells vs. bystander cells. Optimal results were obtained for PLFAER_D_[KLAKLAK]_2_**C**GKRK/Dp44mT equals to 1/3.2. The IC_50_ (drug concentration causing 50% inhibition of cell viability) of this nanocarrier for 4T1 cells was 36 ± 0.03 μg·mL^−1^ and 80 ± 0.03 μg·mL^−1^ for the fibroblast cell line. The absolute concentrations are provided in Table 1.

### 2.6. Prussian Blue Staining

**Figure 5:**
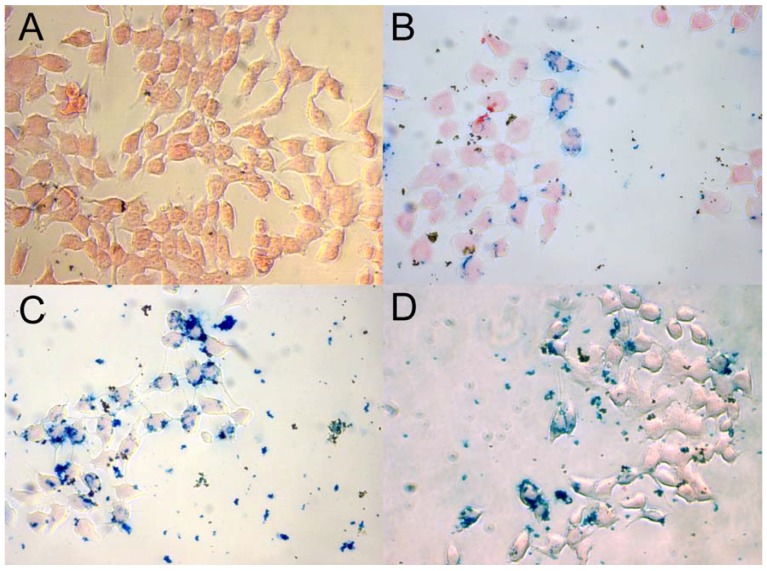
Light microscopy (15×) of Fe/Fe_3_O_4_-uptake by 4T1 cells after 24 h of incubation, followed by a washing procedure, as described in the Methods section. The concentration of all Fe/Fe_3_O_4_-containing nanoparticles in B, C, and D was 5 μg·mL^−1^. (**A**): 4T1 cells, reagent control; (**B**): dopamine-coated Fe/Fe_3_O_4_ nanoparticles; (**C**): Fe/Fe_3_O_4_-Peptide nanocarriers; (**D**): dopamine-coated Fe/Fe_3_O_4_-Peptide/Dp44mT nanocarriers.

**Figure 6:**
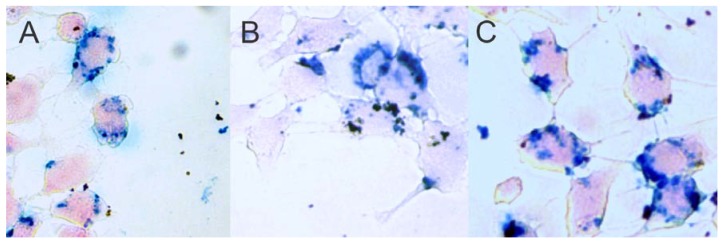
Light microscopy (40×) of Fe/Fe_3_O_4_-uptake by 4T1 cells after 24 h of incubation, followed by a washing procedure, as described in the Methods section. The concentration of all Fe/Fe_3_O_4_-containing nanoparticles in A, B, and C was 5 μg·mL^−1^. (**A**): dopamine-coated Fe/Fe_3_O_4_ nanoparticles; (**B**): Fe/Fe_3_O_4_-Peptide nanocarriers; (**C**): dopamine-coated Fe/Fe_3_O_4_-Peptide/Dp44mT nanocarriers.

From Figure 5B–D and Figure 6it can be discerned that the uptake of Fe/Fe_3_O_4_ by 4T1 cells is heterogeneous. Whereas some cells show very high uptake efficacy, other seem not to be active. This may be related to the cell phase in which contact with dopamine-covered Fe/Fe_3_O_4_ occurs, or it may be caused by the presence of Fe/Fe_3_O_4_-based clusters, as Dynamic Light Scattering indicates. The Fe/Fe_3_O_4_-nanocarriers are found surrounding the cells’ nuclei, which causes ER stress, as discussed in the introduction. The presence of PLFAER_D_[KLAKLAK]_2_CGKRK on the Fe/Fe_3_O_4_-surface enhanced uptake after 24 h, as—qualitatively—can be seen from the comparison of Figure 5B–D.

### 2.7. T1 and T2 Relaxation Times of the Fe/Fe_3_O_4_-derived Nanocarriers

**Figure 7:**
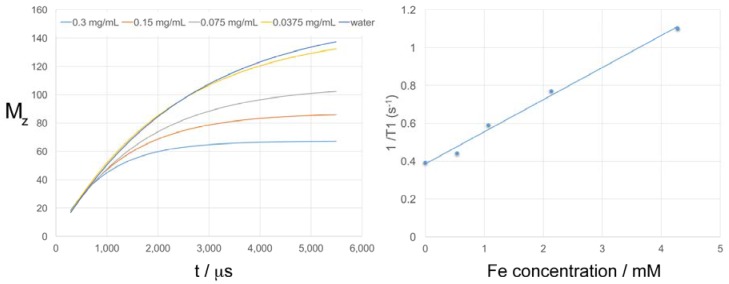
Measurement of T1 relaxation times as a function of dopamine-coated Fe/Fe_3_O_4_-nanocarrier: Bruker Ascend, 14.1 T MRI employing an optimized T1_map_RARE sequence, T_R_ = 2500 ms, and T_E_ = 5.5 ms, slice thickness = 1.5 mm. The plot of the relaxation rate R1 = 1/T1 vs. the iron concentration in mM leads to the relaxivity r1 = 0.285 mM^−1^·s^−1^.

**Figure 8:**
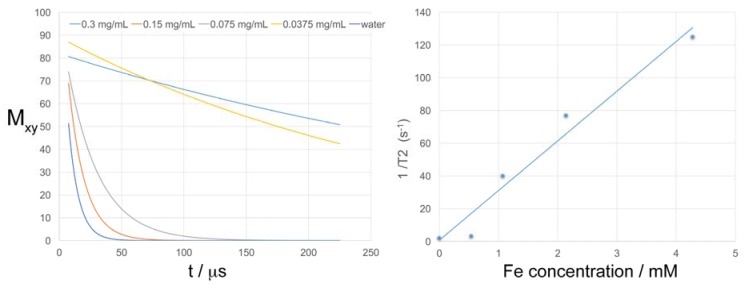
Measurement of T2 relaxation times as a function of dopamine-coated Fe/Fe_3_O_4_-nanocarrier: Bruker Ascend, 14.1 T MRI employing an optimized T2_map_MSME sequence. The plot of the relaxation rate R2 = 1/T2 vs. the iron concentration in mM leads to the relaxivity r2 = 30.0 mM^−1^·s^−1^.

The T1 and T2 relaxation times of dopamine-coated Fe/Fe_3_O_4_-Peptide/Dp44mT (1/3.2) in H_2_O were measured in the ultra-high field of 14.1T by means of T1_map_RARE and T2_map_MSME sequences. The corresponding relaxivities are r1 = 0.285 mM^−1^·s^−1^ and r2 = 30.0 mM^−1^·s^−1^, as shown in Figure 7 and Figure 8.

Iron oxides are more biocompatible than other contrast agents, such as gadolinium- or manganese-based materials. Due to the presence of an Fe(0)-core, the Fe/Fe_3_O_4_-nanoparticles feature a significant T1-relaxity (r1 = 0.285 mM^−1^·s^−1^). Their T2-relaxity r2 is equal to 30.0 mM^−1^·s^−1^, which is smaller than the literature values for typical T2-relaxation agents (Feridex: r2 = 120 mM^−1^·s^−1^, Resovist: r2 = 186 mM^−1^·s^−1^, Combidex: r2 = 65 mM^−1^·s^−1^ at 1.5T) [23]. The number of moles of Fe atoms was obtained via ICP, as described below). It must be noted that at 14.1T the T2-relaxation times are significantly shortened compared to 1.5T, whereas the T1-relaxation times are affected to a lesser extent. The ratio of r2/r1 is the defining parameter with respect to the usefulness of contrast agents for T1-weighted or T2-weighted MR imaging at each magnetic field strength. For the Fe/Fe_3_O_4_-nanoparticles investigated here, r2/r1 = 105 at 14.1T, due to the fact that the average diameter of their Fe(0) core is 24 ± 4 nm and the Fe_3_O_4_ shells are 1.5 ± 0.5 nm thick, which makes them too large to be excellent T1-weighted imaging agents. However, since the materials are nearly monodisperse, they can be used as T1-contrast agents, thus enabling to monitor the drug delivery process in-vivo. Since the Fe_3_O_4_ shells are only 1.5 ± 0.5 nm thick, the Fe/Fe_3_O_4_-nanoparticles are inferior T2-weighted MR-imaging agents compare to Feridex, Resovist, and Combidex [23]. However, they permit monitoring the drug delivery process by means of T2-weighted MR imaging as well. Both, T1- and T2-weighted imaging can be performed at reasonable Fe/Fe_3_O_4_-nanoparticle concentrations (approx. 0.005 g per kg body weight).

## 3. Discussion

**Scheme 2:**
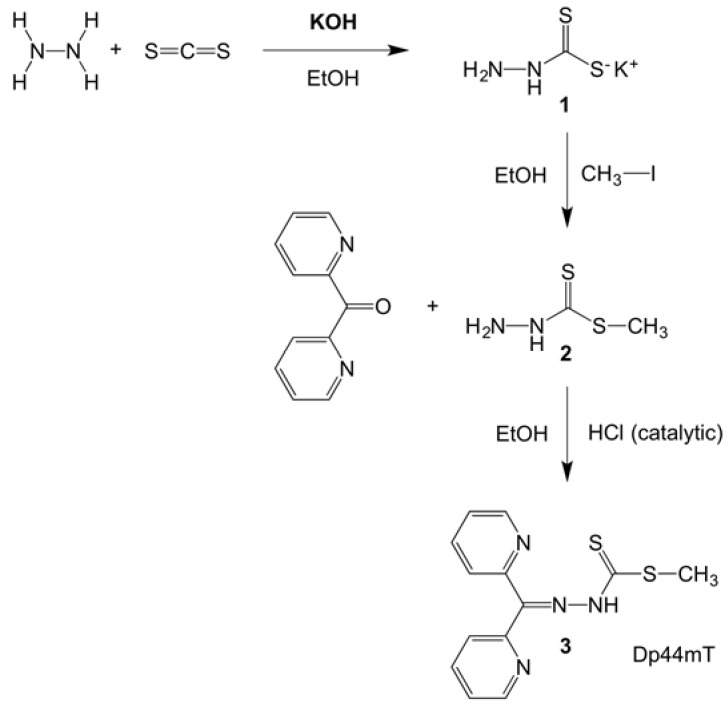
Synthesis of the thiosemicarbazone iron chelator, N′-(Di-pyridin-2-yl-methylene)-hydrazinecarbodithioic acid methylester (Dp44mT).

**Scheme 3:**
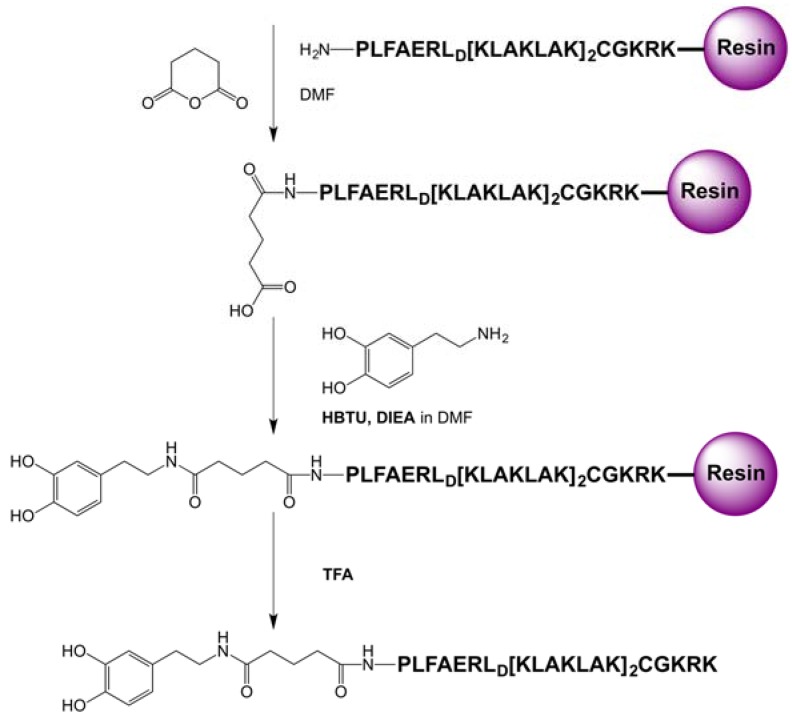
Attachment of a dopamine-unit to PLFAERL_D_[KLAKLAKKLAKLAK]CGKRK. DMF: dimethylformamide; HBTU: *N,N,N′,N′*-Tetramethyl-*O*-(1*H*-benzotriazol-1-yl)uronium hexafluorophosphate; DIEA: *N*,*N*-Diisopropylethylamine; TFA: trifluoroacetic acid (+TIPS: triisopropylsilane).

**Scheme 4:**
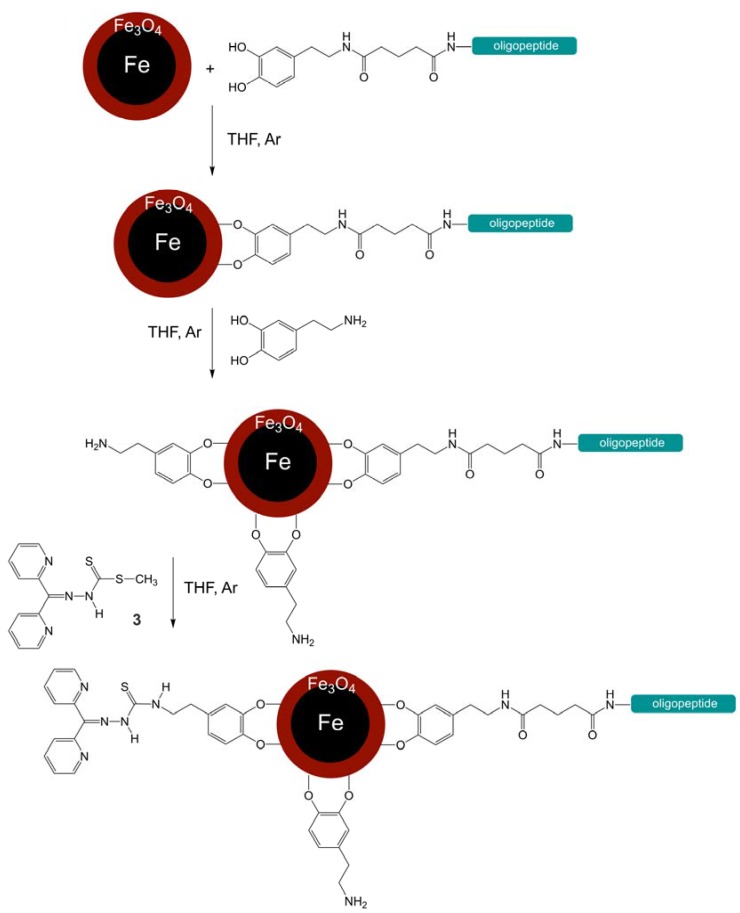
Ligand exchange of the dopamine-glutaric acid-modified peptide sequence PLFAERL_D_[KLAKLAKKLAKLAK]CGKRK against oleylamine and octadecene at the surface of Fe_3_O_4_, followed by additional exchange against dopamine. The latter was then reacted with methyl-2-(di(pyridine-2-yl)methylene)hydrazinecarbodithioate **3** to obtain a Fe/Fe_3_O_4_-nanoparticle- tethered Dp44mT derivative. The structure of Dp44mT is provided in the SI section for comparison.

We have synthesized, purified, and characterized novel dopamine-Fe/Fe_3_O_4_-based nanocarriers that are truly theranostic agents, because they combine at least two treatment modalities with the ability to perform satisfactorily in either T_1_- or T_2_-weighted MR imaging. In opposite to conventional iron-oxide nanoparticles, which are good T_2_-weighted MR imaging agents, the Fe/Fe_3_O_4_-based nanocarriers are suitable T_1_-weighted MR imaging agents as well, due to the presence of a metallic iron(0) core and the small diameter of the core/shell nanoparticles [15,17,23]. We have chosen a hybrid strategy for the synthesis of the peptide sequence- and thiosemicarbazone-tethered carrier nanoparticle, which is summarized in Scheme 2, Scheme 3 and Scheme 4. The Fe/Fe_3_O_4_ core/shell nanoparticles were synthesized and purified by fractionated centrifugation. They were then ligand exchanged first against dopamine-glutaric acid-linked PLFAERL(_D_[KLAKLAKKLAKLAK])CGKRK, and then against dopamine to replace virtually all of the original oleylamine/octadecene ligand shell of the Fe/Fe_3_O_4_-nanoparticles. This was followed by reacting the dopamine ligands at the surface of the Fe_3_O_4_ shell with a reactive thiocarbazone (**3**) to obtain a surface-tethered Dp44mT-derivative. Various ratios of PLFAERL(_D_[KLAKLAKKLAKLAK])CGKRK to thiosemicarbazone were attempted (Table 2). The resulting dopamine-coated Fe/Fe_3_O_4_-nanocarriers were characterized by means of TEM, DLS and zeta potential measurements and ICP (inductively coupled plasma analysis), which permitted the elucidation of the number of peptide sequences and thiosemicarbazones per nanoparticle, making use of the fact that each ligand has only one sulfur atom in its chemical structure. Furthermore, DLS indicated modest clustering of the nanocarriers in PBS, which did not prevent the uptake of the particles, neither by 4T1 cells (metastasizing murine cancer), nor by the murine fibroblast cell line used as control. Light microscopy (40×) of Fe/Fe_3_O_4_-uptake by 4T1 cells after 24 h of incubation, followed by a washing procedure, revealed the uptake mechanism (Figure 6). The dopamine-coated Fe/Fe_3_O_4_ nanoparticles are taken up by endocytosis, followed by endosomal escape (Figure 6A), as previously determined [15]. Clusters of Fe/Fe_3_O_4_-nanocarriers are formed within the endosomes, which then escape together into the cytoplasm. Principally, the same uptake mechanism is discerned for Fe/Fe_3_O_4_-Peptide nanocarriers (Figure 6B). The Fe/Fe_3_O_4_-nanocarriers are again taken up by endosomal uptake and escape. Due to the presence of attached PLFAER_D_[KLAKLAK]_2_**C**GKRK, a fraction of the nanoparticles is being transported to the interior of the cell. C: Dopamine-coated Fe/Fe_3_O_4_-Peptide/Dp44mT nanocarriers follow virtually the same uptake mechanism as Fe/Fe_3_O_4_-Peptide nanocarriers (Figure 6C). It is very likely that the transport processes into the interior of the cell will continue after 48 h (not investigated here), unless the cell is killed.

We have performed MTT assays to measure cell viabilities. Contrary to our original hypothesis, we did not observe a synergy between PLFAER_D_[KLAKLAK]_2_**C**GKRK and the thiosemicarbazone with respect to the IC_50_ values obtained. However, quite unexpectedly, we did observe that the ratio between the determined cell viabilities for 4T1 cancer cells and murine fibroblast (control) cell was a function of PLFAER_D_[KLAKLAK]_2_**C**GKRK to Dp44mT ratio. It was the maximum for a ratio of 1 to 3.2, where a factor of 2.2 was found.

## 4. Conclusions

These results can be regarded as an early step in designing multi-reagent theranostic agents, in which the cytotoxicity vs. cancer cells can be optimized by carefully designed optimization experiments. However, a factor of 2.2 between the determined cell viabilities for 4T1 cancer cells and murine fibroblast (control) cells is not sufficient to suggest starting clinical studies. Further cell, studies will have to be conducted to find nanocarrier compositions capable of killing 4T1 cancer cells with efficacies that are more than 50 times higher than for non-cancerous control cells. These findings will then have to be confirmed in appropriate (syngeneic) mouse experiments.

## 5. Materials and Methods

All chemicals and solvents were purchased either from Aldrich or Fisher. They were at least ACS-grade and used without prior purification. NMR’s were recorded using an 9.7T (400 MHz) Inova NMR Spectrometer (Varian). The T1 and T2 relaxation times were recorded using a Bruker Ascend, 600 MHz, 14.1 T MRI employing optimized T1_map_RARE and T2_map_MSME pulse sequences with T_R_ = 2500 ms, and T_E_ = 5.5 ms, slice thickness = 1.5 mm. All reagents for peptide synthesis were obtained from Peptides International. Mass spectra were recorded using a Voyager DE STR High (Applied Biosystems, Foster City, CA, USA) performance matrix-assisted laser desorption time-of-flight mass spectrometer (MALDI-TOFMS) equipped with a nitrogen laser (337 nm).

### 5.1. Preparation of Core/Shell Fe/Fe_3_O_4_ Magnetic Nanoparticles (MNPs)

The Fe/Fe_3_O_4_ magnetic nanoparticles were synthesized by thermal decomposition of iron pentacarbonyl (Fe(CO)_5_) (10.5 mL) in octadecene (ODE) (300 mL) under argon in the presence of oleylamine (4.5 mL) and hexadecylammonium chloride (HADxHCl) (4.15 g) at 180 °C. This procedure yields highly crystalline iron(0) nanoparticles. When these nanoparticles are exposed to air at room temperature, a thin layer of Fe_3_O_4_ is formed due to the oxidation of the nanoparticle surface. The introduction of the Fe_3_O_4_ shell provides protection against further oxidation and facile surface functionalization of the resulting core/shell Fe/Fe_3_O_4_ nanoparticles. The obtained nanoparticles were washed five times with hexane and ethanol, alternatingly, and then collected by centrifugation (10,000 RPM, 5 min). The nanoparticles were stored in ethanol under argon for further use in this study.

### 5.2. Synthesis of the Dp44mT Derivative (Methyl-2-(di(pyridine-2-yl)methylene)hydrazinecarbo-dithioate)3

The optimized procedure reported here is a modification of the method reported in reference [24]. Potassium hydroxide (8.36 g, 0.14 mol) was dissolved in 90% ethanol (30 mL). This mixture was cooled in an ice bath. To this mixture cold hydrazine hydrate (7.29 mL, 0.14 mol) was added slowly while stirring. Next, cold carbon disulfide (9 mL, 0.14 mol) was added drop-wise under vigorous stirring. The temperature of the reaction mixture was not allowed to rise above 6 °C during the period of carbon disulfide addition. This reaction mixture was reacted for 2 h. To the same reaction mixture cold methyl iodide (9.27 mL, 0.14 mol) was added slowly and further reacted for 30 min. The resulting white product (hydrazinecarbodithioc acid methyl ester **2**) was filtered, washed with cold water, and dried. This product was then recrystallized in hot ethanol. Yield: 91%

^1^H NMR (CDCl_3_-d, δ ppm): 2.66 (s, 3H, CH_3_), 4.15(s, 2H, NH_3_^+^ (protonated nitrogen), 4.66 (s, 2H, NH_2_), 8.11 (s, H, NH_2_^+^CS (protonated nitrogen)), 8.53 (s, H, NHCS), mp (melting point): 79–81 °C.

The ^1^H-NMR spectrum is shown in the SI section (Figure S17).

The methyl ester **2** was then refluxed in the presence of di-2-pyridyl ketone at an equivalence of 3:1, respectively. Di-2-pyridyl ketone (3.01 g, 0.016 mol) was dissolved in 15 mL of ethanol in the presence of a catalytic amount of 1N HCl (2.8 mL). The methyl ester was dissolved in 15 mL of ethanol, added to this mixture, and refluxed at 100 °C for 3 h. After 3 h cold distilled water was added. A yellow colored precipitate formed immediately. This precipitate was filtered, and washed with cold water, and dried in a desiccator over P_2_O_5_. The resulting product was recrystallized in hot ethanol. The resulting yellow colored needle shaped crystals were filtered, washed with cold ethanol and stored in a desiccator over P_2_O_5_. Yield: 82%.

^1^H NMR (CDCl3, δ ppm): 2.67 (s, 3H, CH_3_); 7.52 (s, H, NH); 8.81 (d, H3), 8.62 (d, H8), 8.06 (d, H6), 7.84 (m, H5 & 8), 7.73 (d, H7), 7.42 (m, H4), 7.37 (m, H9) of the Ar–H.

^13^C NMR (CDCl_3_, δ ppm, J, Hz): 17.70 (CH_3_); 124.08 (C10), 124.46 (C5), 124.69 (C12), 127.56 (C7), 137.33 (C6 & 11), 142.55 (C13), 148.30 (C8), 148.62 (C9), 151.31 (C4) of the Ar–C; 155.68 (C=N); 202.44 (C=S). mp: 150–153°C. The ^1^H-NMR and ^13^C-NMR spectra are shown in the SI section (Figures S18–S20).

### 5.3. Synthesis of PLFAERL_D_[KLAKLAK]_2_CGKRK

The peptide sequence PLFAERL_D_[KLAKLAKKLAKLAK]CGKRK was synthesized by solid phase peptide synthesis, as previously described [18,19,25]. In short, the peptide was synthesized on a lysine preloaded trityl resin by means of solid-phase supported synthesis from the C-terminus to the N-terminus.

### 5.4. Synthesis of the Dopamine Peptide Ligand

The resin with the peptide (150 mg, 0.075 mmol) was incubated with 5 mL of DCM for 20 min. The DCM was filtered off and the resin was swirled with 2.5 mL of anhydrous DMF for 1 min, which was then filtered off. Then the peptide sequence PLFAERL(_D_[KLAKLAKKLAKLAK])CGKRK was F_moc_-deprotected by reaction with 4 mL of 20% piperidine: DMF solution for 1 min while still on resin. The resin was filtered off. The deprotection step was repeated during 10 min, followed by filtration. The resin was washed 5 times with 2.5 mL of DMF and filtered off each time. The resin with peptide sequence was then reacted with glutaric anhydride (25.67 mg, 0.225 mmol) for 3 h. This step was repeated twice. This glutaric acid-attached peptide sequence was reacted with dopamine hydrochloride (14.22 mg, 0.075 mmol) at a molar ratio of 1:1 and HBTU (82.40 mg, 0.217 mmol) at a molar ratio of 1:2.9 in 6 mL of DIEA (0.5 mmol) in DMF coupling solution for 30 min and then filtered off. This step was repeated once. The resin was washed 4 times with 2.5 mL of DMF for 30 s each and was filtered off. It was then washed 5 times with 2.5 mL of DCM for 30 s each and was filtered off. Next the dopamine peptide ligand was cleaved from the resin using 2 mL of the cocktail of 1.9 mL TFA, 0.05 mL TIPS and 0.05 mL distilled water, which was swirled for 3 h. The cleaved dopamine peptide ligand was precipitated in 15 mL of cold diethyl ether and was removed by centrifugation (15,000 RMP for 30 min). The precipitated ligand was washed two more times with 15 mL of cold ether. The dopamine-glutaric acid-modified peptide sequence was centrifuged off and the ligand was dried and stored under argon in a −80 °C freezer. The reaction is shown in Scheme 3.

### 5.5. HPLC Analysis of the Peptide PLFAERL_D_[KLAKLAKKLAKLAK]CGKRK

The peptide sequence PLFAERL_D_[KLAKLAKKLAKLAK]CGKRK was analyzed using the Waters 1525- Binary HPC pump instrument and Kinetex 5 μ XB- C18 100 A size 250 × 4.6 mm reverse phase column. The peptide (2.5 mg) was dissolved in equal amounts of 0.2% TFA in H_2_O (125 μL) and acetonitrile (125 μL), injection volume: 40 μL. The peptide was separated from by-products formed during solid-phase supported synthesis using 0.2% TFA in H_2_O as the aqueous solvent and acetonitrile as the organic solvent. The gradient is provided in the SI section, together with the HLPC chromatogram. (Figure S1).

### 5.6. Ligand Exchange of Fe/Fe_3_O_4_ Nanoparticles with the Dopamine Peptide Sequence and Free Dopamine

The oleylamine and octadecyl coated Fe/Fe_3_O_4_ nanoparticles (500 mg) were reacted with dopamine-glutaric acid-modified PLFAERL_D_[KLAKLAKKLAKLAK]CGKRK (50 mg) at a molar ratio of 10:1 in 10 mL of anhydrous THF (Tetrahydrofuran) under argon for 12 h at RT. The reaction mixture was centrifuged (10,000 RPM, 5 min), and the supernatant was removed. In the next step the nanoparticles were then reacted with an excess amount of dopamine (150 mg) in 10 mL of THF under argon for 12 h at RT. The reaction mixture was centrifuged (10,000 RPM, 5 min), and the supernatant was removed. The nanoparticles were washed 5 times with 5 mL of DCM each. The nanoparticles were stored in DCM for future use. The reaction is shown in Scheme 4. A second batch of nanoparticles was synthesized with just dopamine as ligand, following, principally, the same procedure. This batch was used as a control in the cell experiments.

Coupling of compound **3** to the primary amine group of dopamine at the Fe_3_O_4_-surface was performed in different ratios of compound **3** to free dopamine at the surface. Molar rations of 1:10, 1:5, 1:1, 5:1, and 10:1, respectively, were chosen. The coupling reaction was performed in anhydrous DMF. A two-fold molar excess of EDC, HOBT, with respect to compound **3**, was used. The molar ratio of DIEA to compound **3** was 2.5:1. The reaction was performed for 4d at 300 K under argon. After completion, the resulting Fe/Fe_3_O_4_-nanocarriers were harvested by means of centrifugation (10,000 RPM, 5 min) and washed five times with methanol and once with H_2_O. After drying in high vacuum, the nanocarriers were stored at RT under Ar.

### 5.7. Dynamic Light Scattering (DLS) and Zeta Potential Measurements

DLS and Zeta-potential measurements were performed using ZetaPALS (Brookhaven Instruments Corp., Holtsville, NY, USA) 1.0 mg of nanoparticles were sonicated for 15 min in 1 mL of distilled water. A volume of 75 μL of this solution was diluted with 3 mL of distilled water. DLS and zeta potential measurements were recorded of this dispersion.

### 5.8. Transmission Electron Microscopy (TEM)

The size of the nanoparticles was determined by TEM, which was performed in the Microscopy and Analytical Imaging Laboratory of the University of Kansas. Samples were prepared by suspending the Fe/Fe_3_O_4_-nanocarriers in DCM, followed by ultrasonication for 15 min. The samples were placed onto copper mesh grids with lacey carbon films. The wet grids were allowed to air-dry for several minutes prior to being examined by means of TEM. The nanoparticle’s sizes and morphology were examined by bright-field and dark-field transmission electron microscopy (TEM) using an FEI Technai G_2_ transmission electron microscope (Hillsboro, OR, USA) at an electron acceleration voltage of 200 kV. High resolution images were captured using a standardized, normative electron dose and a constant defocus value from the carbon-coated surfaces. Energy dispersive X-ray spectroscopy (EDS) was carried out using an EDAX detector.

### 5.9. Inductively Coupled Plasma (ICP) Measurements to Determine the Iron and Sulfur Content of the Fe/Fe_3_O_4_ Nanocarriers

ICP Measurements were performed using a ICP (Varian 720-ES Inductively Coupled Plasma-Optical Emission Spectrometer, Varian: Palo Alto, CA, USA). The calculation of the accessible surface area can be found in the SI section. ICP measurements of both, the dopamine-coated Fe/Fe_3_O_4_-nanoparticles (control) and the Fe/Fe_3_O_4_-nanocarriers were performed, comprising all molar ratios of thiosemicarbazone to the nanoparticles (1:10, 1:5, 1:1, 5:1 and 10:1). To determine the iron content, 2.0 mg of the nanocarriers was weighed and digested in 1.0 mL of 10% HCl. Then, 100 μL of concentrated sample were diluted to a total volume of 5 mL with 10% HCl. To determine the sulfur content, 6.0 mg of the nanosystem was weighed and digested in 3.0 mL of 10% HCl. A standard concentration series of iron and sulfur was prepared using iron nitrate (Fe(NO_3_)_3_ 1000 mg/L) and sulfuric acid (H_2_SO_4_ 1000 mg/L) in 10% HCl (Table 3).

### 5.10. Cell Viability Test Measured by MTT Assay

Cell viabilities were determined for both, the 4T1 murine breast cancer cell line and a normal (healthy) murine fibroblast cell line, after incubation with the dopamine-coated Fe/Fe_3_O_4_-nanoparticles, and all Fe/Fe_3_O_4_-nanocarriers featuring various ratios of attached therapeutic peptides and the Dp44mT-derivative (1:10, 1:5, 1:1, 5:1 and 10:1). The test was performed in 96 well plates. The plates were divided into three columns of 4 replicas each for the control, 24 h and 48 h cell viabilities, which were tested for concentrations of 0 μg/mL (control), 10 μg/mL, 50 μg/mL, 100 μg/mL for all ratios of therapeutic peptides vs. Dp44mT-derivative. The 4T1 murine breast cancer cells were cultured in RPMI (Roswell Park Memorial Institute) medium with 10% FBS (Fetal bovine serum) and 1× penicillin-streptomycin in a 37 °C humidified incubator with 5% CO_2_ atmosphere. After 24 h the growth medium was removed and 75 μL of fresh medium was added to the wells. To the 24 h cells 7.5 μL of MTT reagent (3-(4,5-Dimethylthiazol- 2-yl)-2,5-diphenyltetrazolium bromide) was added and incubated for 4 h. After 4h 75 μL of MTT buffer solution was added to the 24 h wells to dissolve the formazan crystals overnight. After overnight incubation, UV/Vis absorption spectra were recorded in a range from 550 nm to 690 nm. The obtained UV/Vis absorption peaks were integrated and compared with a calibration curve. The same procedure was carried out for the fibroblast cell line except the growth medium was DMEM (Dulbecco’s Modified Eagle’s Medium). Cell toxicity data were analyzed as follows: (1) For each well the absorption at λ = 690 nm was subtracted from the absorption at λ = 540 nm; (2) The average and standard deviation was calculated for each series of repetitions (*n* = 4 to 6); (3) The mean values of the blanks (solvent controls) were calculated; (4) The *Relative Inhibition Activity* is expressed as:

$$\%\;Inhibition=100-\left[\frac{\left[mean\;value\;of\;A\left(540nm\right)-A\left(690nm\right)\;of\;sample\right]\;\times \;100}{\left[mean\;value\;of\;A\left(540nm\right)-A\left(690nm\right)\;of\;solvent\;control\right]}\right]$$

The cell toxicity curve was obtained by plotting the % inhibition of activity vs. the concentration of the Fe/Fe_3_O_4_-based nanocarriers. The IC_50_ values (half maximal inhibitory concentration) were calculated from the following plot. The IC_50_ values are obtained from the intercept with the x-axis [26].

$$Log\;\left[\frac{\left(\%\;inhibition\right)}{100-\%\;inhibition}\right]\;vs.\;concentration$$

### 5.11. Test for Cell Uptake of Nanoparticles by Prussian Blue Staining

Fe/Fe_3_O_4_-nanocarrier uptake tests uptake were performed using dopamine-coated Fe/Fe_3_O_4_-nanoparticles, as well as Fe/Fe_3_O_4_-nanocarriers featuring a 5:1 ratio of Dp55mT-derivative to therapeutic peptide sequence. Nanoparticle dispersions were prepared in the respective growth media. The following concentrations were tested; 0 μg/mL (control) 5 μg/mL, and 10 μg/mL. The test was conducted in duplicate. Murine breast cancer cells 4T1, were grown in 24 well plates. The cells were grown in RPMI with 10% FBS and 1× penicillin-streptomycin in a 37 °C humidified incubator with 5% CO_2_ atmosphere for 24 h. After 24 h the medium was removed and 500 μL of the selected nanoparticle dispersion was added. 500 μL RPMI medium was added as control. The wells were incubated for 24 h. After 24 h the staining procedure was carried out. The cells were washed twice with 500 µL RPMI and twice with 1× PBS (Phosphate buffered saline). Then, 500 μL of 10% NBF (neutral buffered formalin) was added and was allowed to stand for 10 min for fixation to occur. The cells were washed three times with 500 μL of 1× PBS. Then, equal amounts of 4% potassium ferrocyanide and 4% HCl (500 μL each) were added to the wells. Again, 10 min for reaction were allowed. This step was repeated. Then the wells were rinsed three times with 500 μL distilled water. Next, the cells were stained with 200 μL of nuclear fast red for 5 min and washed four times with 500 μL distilled water. The cells were stored in 500 μL PBS and were observed under a light microscope (Axiovert 40 CFL (Zeiss: Oberkochen, Germany) with brightfield and phase contrast illumination, a ProgRes C3 Cool camera (Jenoptik: Jena, Germany) with ProgRes Capture Pro 2.10.0.0 software).

## Figures and Tables

**Table 1:** Percentage of Iron in Fe/Fe_3_O_4_-Nanocarriers, as well as Labeling Degree with the Therapeutic Peptide PLFAER_D_[KLAKLAK]_2_**C**GKRK and Dp44mT-Derivative.

Nanoparticle System	Fe Percentage of the Nanoparticles	S Percentage of the Nanoparticles	Moles of S Per 1.0 mg of Nanoparticle	Ratio of Therapeutic Peptide/Dp44mT
Dop-Fe/Fe_3_O_4_^1^	71.0%	/	/	/
Dop-Fe/Fe_3_O_4_ Peptide	72.0%	0.021%	6.50 × 10^−6^	/
Dop-Fe/Fe_3_O_4_ Peptide/Dp44mT (10:1) ^2^	70.6%	0.040%	1.24 × 10^−5^	1:1.1
Dop-Fe/Fe_3_O_4_ Peptide/Dp44mT (5:1) ^2^	72.6%	0.087%	2.73 × 10^−5^	1:3.2
Dop-Fe/Fe_3_O_4_ Peptide/Dp44mT (1:1) ^2^	72.8%	0.58%	1.86 × 10^−4^	1:27
Dop-Fe/Fe_3_O_4_ Peptide/Dp44mT (1:5) ^2^	72.8%	1.56%	4.87 × 10^−4^	1:74
Dop-Fe/Fe_3_O_4_ Peptide/Dp44mT (1:10) ^2^	71.9%	2.06%	6.77 × 10^−4^	1:103

^1^ Dop = dopamine; ^2^ Attempted ratio of therapeutic peptide and Dp44mT-derivative The relative error of the ICP measurements is ± 5%.

**Table 2:** Hydrodynamic Diameter of the Fe/Fe_3_O_4_-Nanocarriers in Dependence of the Labeling Degree with the Therapeutic Peptide PLFAER_D_[KLAKLAK]_2_**C**GKRK and Dp44mT-Derivative. Typical polydispersities range from 0.20 to 0.30.

Nanoparticle System	Hydrodynamic Diameter of Nanoparticles (nm)	Zeta Potential (mV)	Ratio of Therapeutic Peptide/Dp44mT
Dop-Fe/Fe_3_O_4_^1^	475 ± 20	+30.5 ± 2	/
Dop-Fe/Fe_3_O_4_ Peptide	600 ± 35	+35.3 ± 3	/
Dop-Fe/Fe_3_O_4_ Peptide/Dp44mT (10:1) ^2^	505 ± 27	+24.4 ± 3	1/1.1
Dop-Fe/Fe_3_O_4_ Peptide/Dp44mT (5:1) ^2^	430 ± 45	+14.8 ± 3	1/3.2
Dop-Fe/Fe_3_O_4_ Peptide/Dp44mT (1:1) ^2^	530 ± 45	+37.1 ± 2	1/27
Dop-Fe/Fe_3_O_4_ Peptide/Dp44mT (1:5) ^2^	425 ± 50	+38.7 ± 3	1/74
Dop-Fe/Fe_3_O_4_ Peptide/Dp44mT (1:10) ^2^	450 ± 30	+32.1 ± 3	1/103

^1^ Dop = dopamine; ^2^ Attempted ratio of therapeutic peptide and Dp44mT-derivative.

**Table 3:** Standard concentration series for iron and sulfur content determination by means of ICP.

Fe-conc. (mg/L)	S-conc. (mg/L)
0	0
10	5
20	10
40	15
60	20

Fe(NO_3_)_3_ and H_2_SO_4_ in 10% aqueous HCl.

## Supplementary Materials

The following are available online at www.mdpi.com/2079-4983/8/3/23/s1: Figure S1: HPLC of PLFAERL(_D_[KLAKLAKKLAKLAK]) CGKRK. At *t* = 51.62 min the peptide was detected and characterized by MALDI-TOF (Figure S2). Its purity was determined to 94.6%, assuming that all intermediates have the same UV-absorption at 200 nm; Figure S2: MALDI-TOF of PLFAERL(_D_[KLAKLAKKLAKLAK])CGKRK (Voyager DE STR); Figure S3: Dynamic Light Scattering (DLS) characterization of dopamine-coated Fe/Fe_3_O_4_ in phosphate-buffered saline (PBS), see Table 2; Figure S4: DLS characterization of PLFAER_D_[KLAKLAK]_2_**C**GKRK-tethered dopamine-coated Fe/Fe_3_O_4_ in PBS (Dop-Fe/Fe_3_O_4_ Peptide), see Table 2; Figure S5: DLS characterization of PLFAER_D_[KLAKLAK]_2_**C**GKRK- and Dp44mT-derivative- tethered dopamine-coated Fe/Fe_3_O_4_ in PBS (Dop-Fe/Fe_3_O_4_ Peptide/Dp44mT (1:10)), ratio of Peptide to Dp44mT = 1/103, see Table 2; Figure S6: DLS characterization of PLFAER_D_[KLAKLAK]_2_**C**GKRK- and Dp44mT-derivative- tethered dopamine-coated Fe/Fe_3_O_4_ in PBS (Dop-Fe/Fe_3_O_4_ Peptide/Dp44mT (1:5)), ratio of Peptide to Dp44mT = 1/74, see Table 2; Figure S7: DLS characterization of PLFAER_D_[KLAKLAK]_2_**C**GKRK- and Dp44mT-derivative- tethered dopamine-coated Fe/Fe_3_O_4_ in PBS (Dop-Fe/Fe_3_O_4_ Peptide/Dp44mT (1:1)), ratio of Peptide to Dp44mT = 1/27, see Table 2. Figure S8: DLS characterization of PLFAER_D_[KLAKLAK]_2_**C**GKRK- and Dp44mT-derivative- tethered dopamine-coated Fe/Fe_3_O_4_ in PBS (Dop-Fe/Fe_3_O_4_ Peptide/Dp44mT (5:1)), ratio of Peptide to Dp44mT = 1/3.2, see Table 2; Figure S9: DLS characterization of PLFAER_D_[KLAKLAK]_2_**C**GKRK- and Dp44mT-derivative- tethered dopamine-coated Fe/Fe_3_O_4_ in PBS (Dop-Fe/Fe_3_O_4_ Peptide/Dp44mT (10:1)), ratio of Peptide to Dp44mT = 1/1.1, see Table 2; Figure S10: Zeta potential of dopamine-coated Fe/Fe_3_O_4_ in PBS; Figure S11: Zeta potential of of PLFAER_D_[KLAKLAK]_2_**C**GKRK-tethered dopamine-coated Fe/Fe_3_O_4_ in PBS (Dop-Fe/Fe_3_O_4_ Peptide), see Table 2; Figure S12: Zeta potential of PLFAER_D_[KLAKLAK]_2_**C**GKRK- and Dp44mT-derivative- tethered dopamine-coated Fe/Fe_3_O_4_ (Dop-Fe/Fe_3_O_4_ Peptide/Dp44mT (1:10)), ratio of Peptide to Dp44mT = 1/103, see Table 2; Figure S13: Zeta potential of PLFAER_D_[KLAKLAK]_2_**C**GKRK- and Dp44mT-derivative- tethered dopamine-coated Fe/Fe_3_O_4_ (Dop-Fe/Fe_3_O_4_ Peptide/Dp44mT (1:5)), ratio of Peptide to Dp44mT = 1/74, see Table 2; Figure S14: Zeta potential of PLFAER_D_[KLAKLAK]_2_**C**GKRK- and Dp44mT-derivative- tethered dopamine-coated Fe/Fe_3_O_4_ (Dop-Fe/Fe_3_O_4_ Peptide/Dp44mT (1:1)), ratio of Peptide to Dp44mT = 1/27, see Table 2; Figure S15: Zeta potential of PLFAER_D_[KLAKLAK]_2_CGKRK- and Dp44mT-derivative- tethered dopamine-coated Fe/Fe_3_O_4_ (Dop-Fe/Fe_3_O_4_ Peptide/Dp44mT (5:1)), ratio of Peptide to Dp44mT = 1/3.2, see Table 2; Figure S16: Zeta potential of PLFAER_D_[KLAKLAK]_2_**C**GKRK- and Dp44mT-derivative- tethered dopamine-coated Fe/Fe_3_O_4_ (Dop-Fe/Fe_3_O_4_ Peptide/Dp44mT (10:1)), ratio of Peptide to Dp44mT = 1/1.1, see Table 2; Figure S17: ^1^HNMR of Hydrazinecarbodithioic acid methylester; Figure S18: ^1^HNMR of the thiosemicarbazone iron chelator (compound **3**; Figure S19: ^1^HNMR of the aryl hydrogens of the thiosemicarbazone iron chelator, N′-(Di-pyridin-2yl-methylene)- hydrazinecarbodithioic acid methylester, Figure S20: ^13^CNMR of the thiosemicarbazone iron chelator N′-(Di-pyridin-2yl-methylene)-hydrazinecarbodithioic acid methylester. ^13^C NMR (CDCl_3_, δ ppm, J, Hz): 17.70 (CH_3_); 124.08 (C10), 124.46 (C5), 124.69 (C12), 127.56 (C7), 137.33 (C6 & 11), 142.55 (C13), 148.30 (C8), 148.62 (C9), 151.31 (C4) of the Ar–C; 155.68 (C=N); 202.44 (C=S).

## Abbreviations

AMPK: 5′ AMP-activated protein kinase; ATF6: activating transcription factor 6; PERK: kinase R (PKR)-like endoplasmic reticulum kinase; eIF3α: eukaryotic initiation factor 3α; IRE1α: Inositol-requiring protein 1α; IC_50_: inhibitory concentration at 50% deactivation; PBS: phosphate-buffered saline, HBTU: *N,N,N′,N′*-Tetramethyl-*O-*(1*H*-benzotriazol-1-yl)uronium hexafluorophosphate, DIEA: N,N-Diisopropylethylamine, DMF: dimethylformamide, DCM: dichloromethane, TFA: trifluoroacetic acid, TIPS: triisopropylsilane, MTT: 3-(4,5-dimethylthiazol-2-yl)-2,5-diphenyltetrazolium bromide.
