# Supplementary material for: Synergy of Iron Chelators and Therapeutic Peptide Sequences Delivered via a Magnetic Nanocarrier
Source: J Funct Biomater. 2017 Jun 26;8(3):23. doi: 10.3390/jfb8030023 (PMC5618274; doi:10.3390/jfb8030023)
Supplement: Supplementary file 1 [file jfb-08-00023-s001.docx]

Synergy of Iron Chelators and Therapeutic Peptide Sequences

Gayani S. Abayaweera ^1^, Hongwang Wang ^1^, Tej B. Shrestha ^2^, Jing Yu ^1^, Kyle Angle ^1^, Prem Thapa ^3^, Aruni P. Malalasekera ^1^, Leila Maurmann ^1^, Deryl L. Troyer ^2^, and Stefan H. Bossmann ^1,^*

^1^ Kansas State University, Department of Chemistry, Manhattan, KS 66041, USA;

^2^ Kansas State University, Department of Anatomy & Physiology, Manhattan, KS 66041, USA;

^3^ University of Kansas, Microscopy and Analytical Imaging Laboratory, Lawrence, KS 66045. USA

***** Correspondence: sbossman@ksu.edu; Tel.: +001-785-532-6817


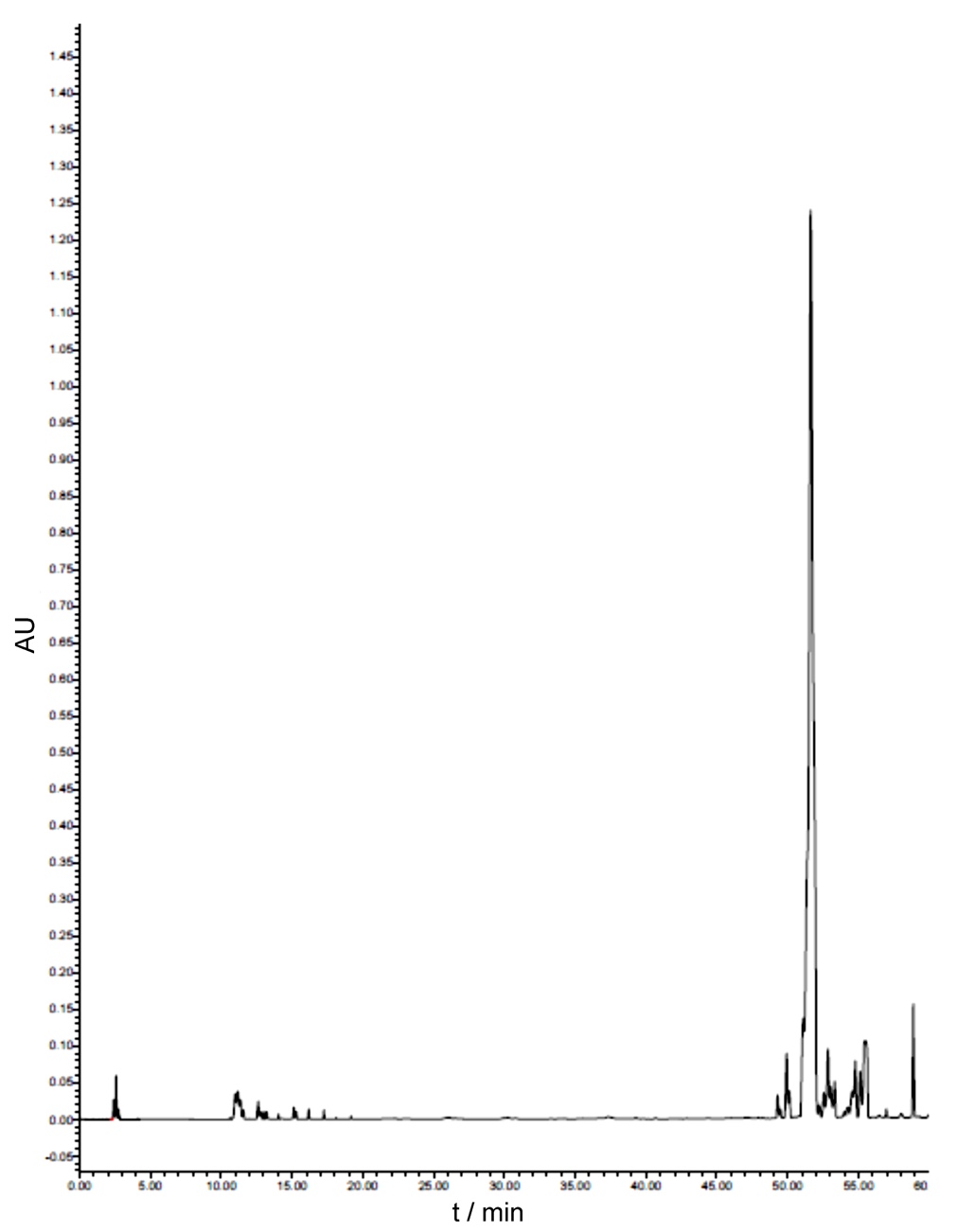


**Figure S1**. HPLC of PLFAERL(_D_[KLAKLAKKLAKLAK]) CGKRK. At t = 51.62 min the peptide was detected and characterized by MALDI-TOF (Figure S2). Its purity was determined to 94.6 %, assuming that all intermediates have the same UV-absorption at 200nm.


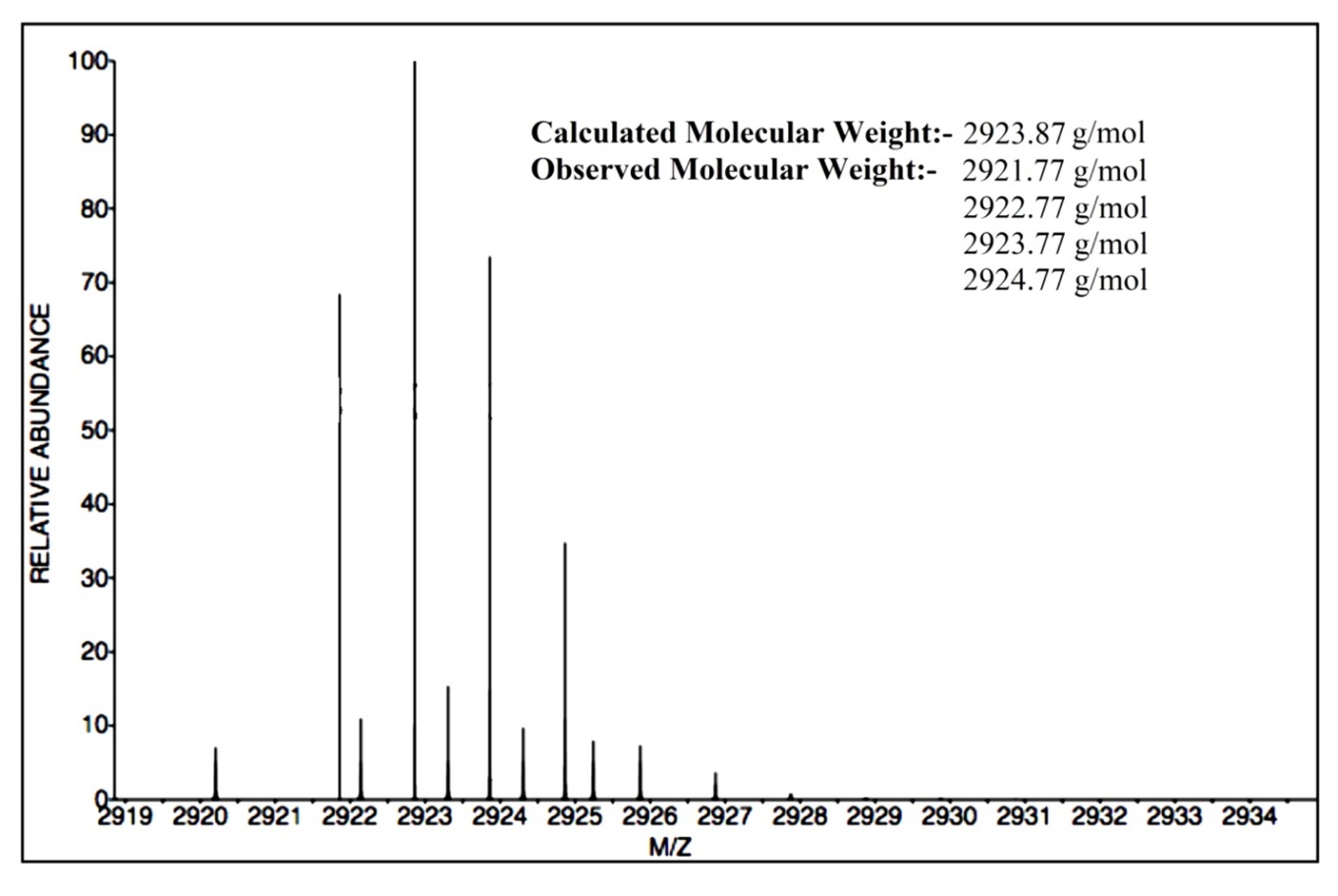


**Figure S2.** MALDI-TOF of PLFAERL(_D_[KLAKLAKKLAKLAK])CGKRK (Voyager DE STR).


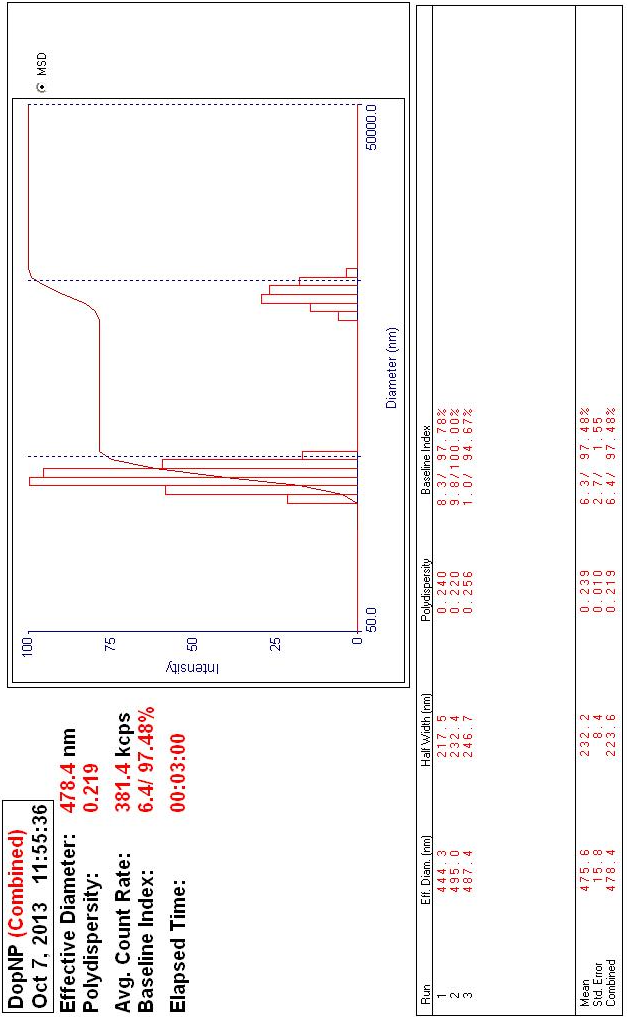


**Figure S3.** Dynamic Light Scattering (DLS) characterization of dopamine-coated Fe/Fe_3_O_4_ in phosphate-buffered saline (PBS), see Table 2.


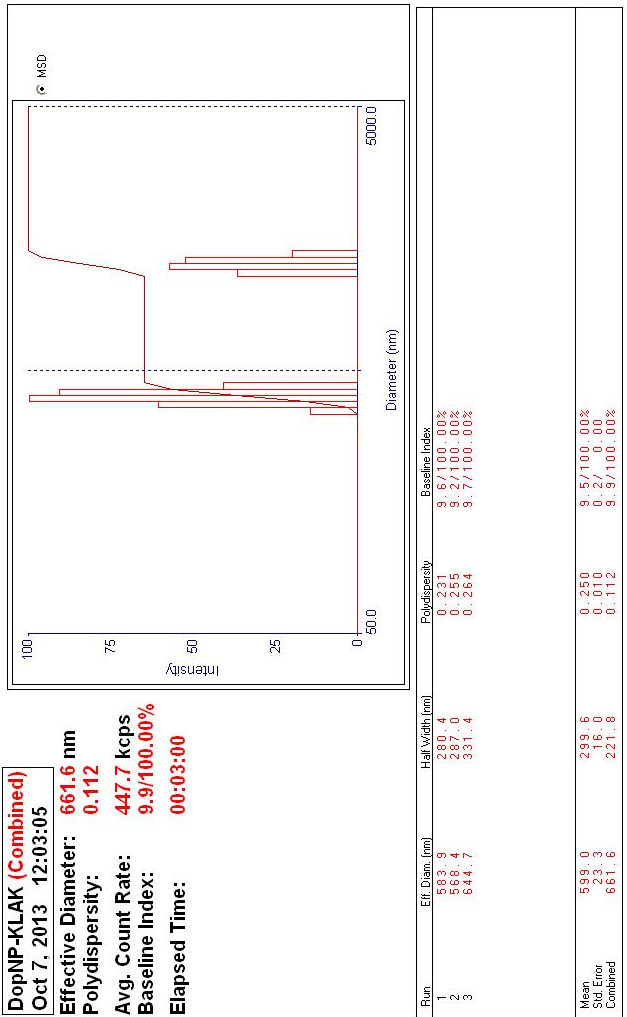


**Figure S4.** DLS characterization of PLFAER_D_[KLAKLAK]_2_**C**GKRK-tethered dopamine-coated Fe/Fe_3_O_4_ in PBS (Dop-Fe/Fe_3_O_4_ Peptide), see Table 2.


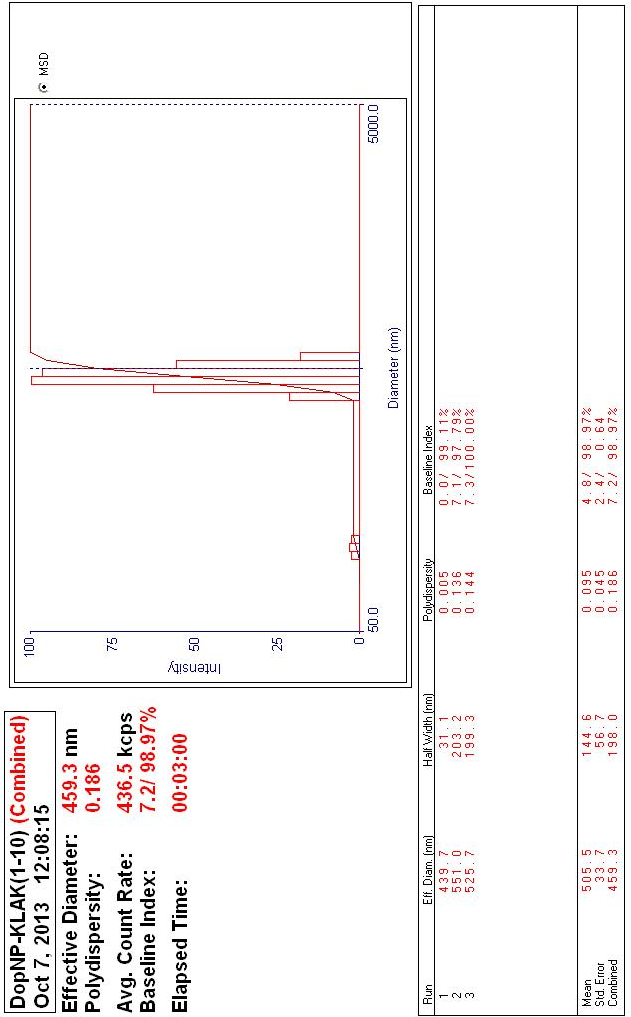


**Figure S5.** DLS characterization of PLFAER_D_[KLAKLAK]_2_**C**GKRK- and Dp44mT-derivative- tethered dopamine-coated Fe/Fe_3_O_4_ in PBS (Dop-Fe/Fe_3_O_4_ Peptide/Dp44mT (1:10)), ratio of Peptide to Dp44mT = 1/103, see Table 2.


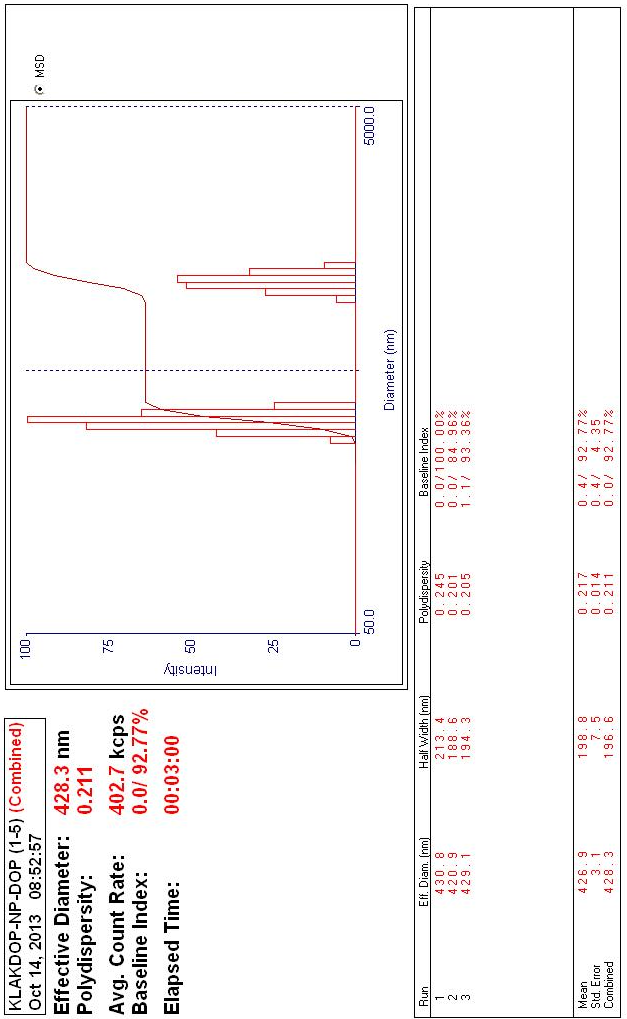


**Figure S6.** DLS characterization of PLFAER_D_[KLAKLAK]_2_**C**GKRK- and Dp44mT-derivative- tethered dopamine-coated Fe/Fe_3_O_4_ in PBS (Dop-Fe/Fe_3_O_4_ Peptide/Dp44mT (1:5)), ratio of Peptide to Dp44mT = 1/74, see Table 2.


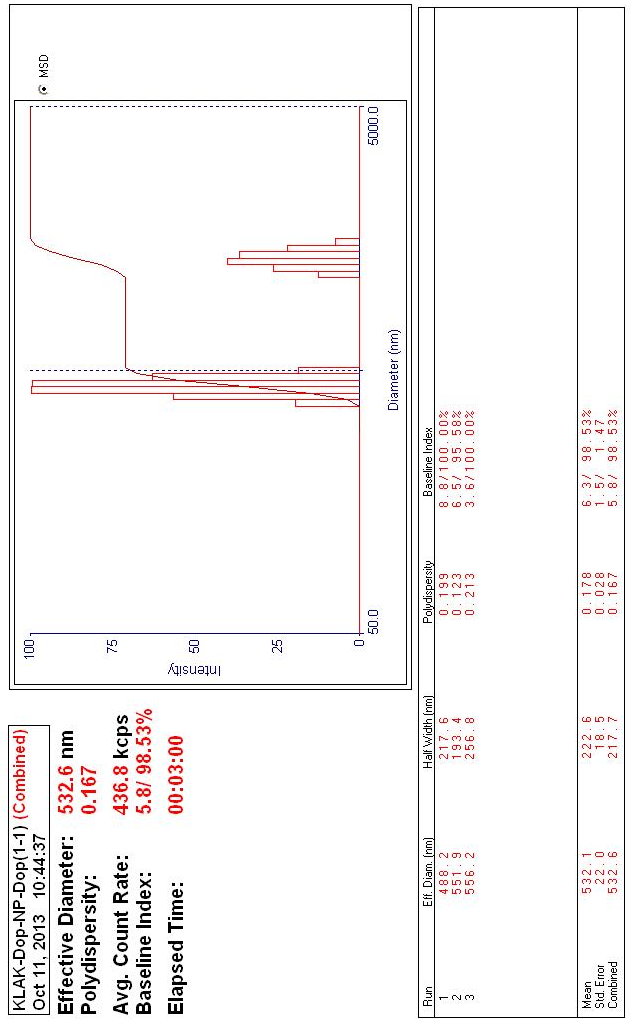


**Figure S7.** DLS characterization of PLFAER_D_[KLAKLAK]_2_**C**GKRK- and Dp44mT-derivative- tethered dopamine-coated Fe/Fe_3_O_4_ in PBS (Dop-Fe/Fe_3_O_4_ Peptide/Dp44mT (1:1)), ratio of Peptide to Dp44mT = 1/27, see Table 2.


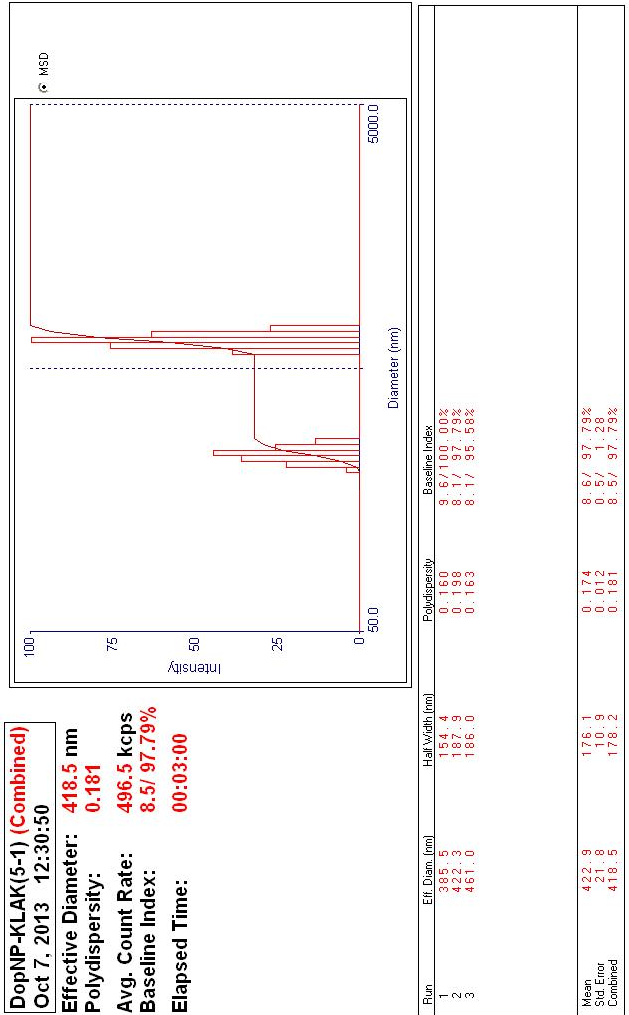


**Figure S8.** DLS characterization of PLFAER_D_[KLAKLAK]_2_**C**GKRK- and Dp44mT-derivative- tethered dopamine-coated Fe/Fe_3_O_4_ in PBS (Dop-Fe/Fe_3_O_4_ Peptide/Dp44mT (5:1)), ratio of Peptide to Dp44mT = 1/3.2, see Table 2.


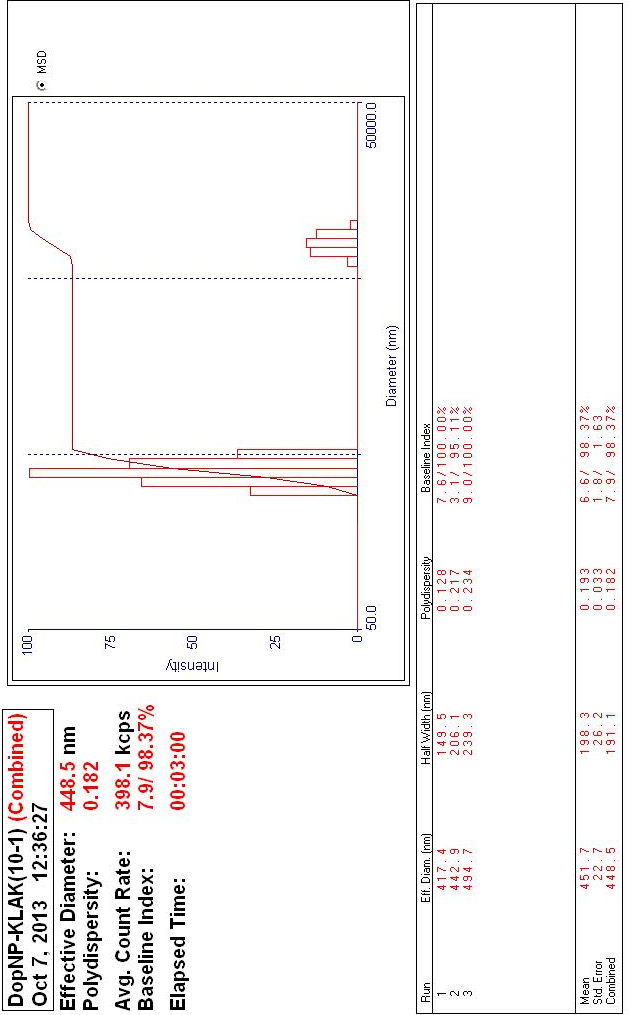


**Figure S9.** DLS characterization of PLFAER_D_[KLAKLAK]_2_**C**GKRK- and Dp44mT-derivative- tethered dopamine-coated Fe/Fe_3_O_4_ in PBS (Dop-Fe/Fe_3_O_4_ Peptide/Dp44mT (10:1)), ratio of Peptide to Dp44mT = 1/1.1, see Table 2.


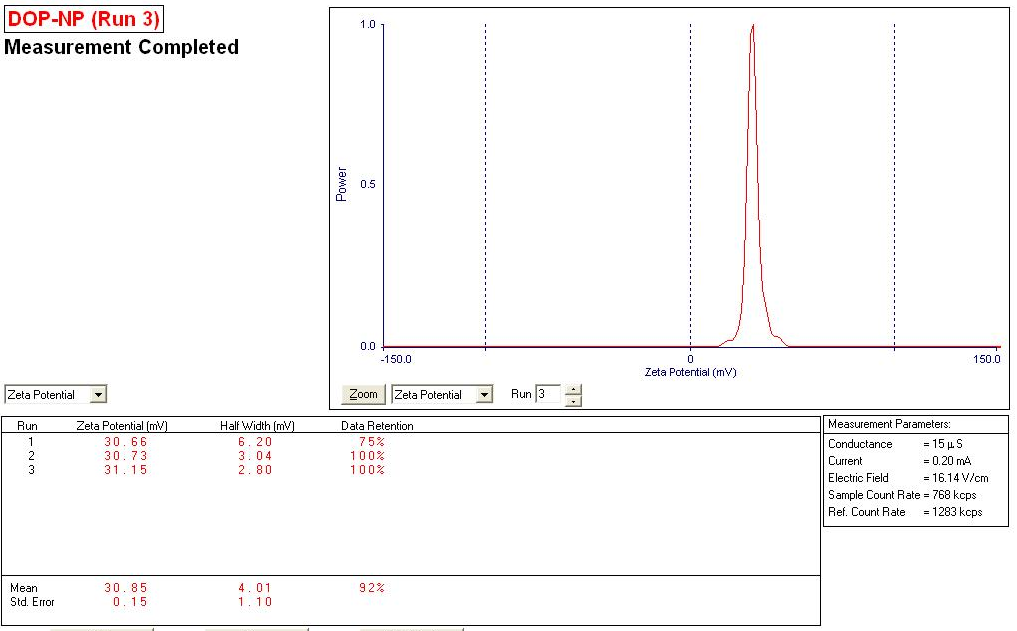


**Figure S10.** Zeta potential of dopamine-coated Fe/Fe_3_O_4_ in PBS.

**
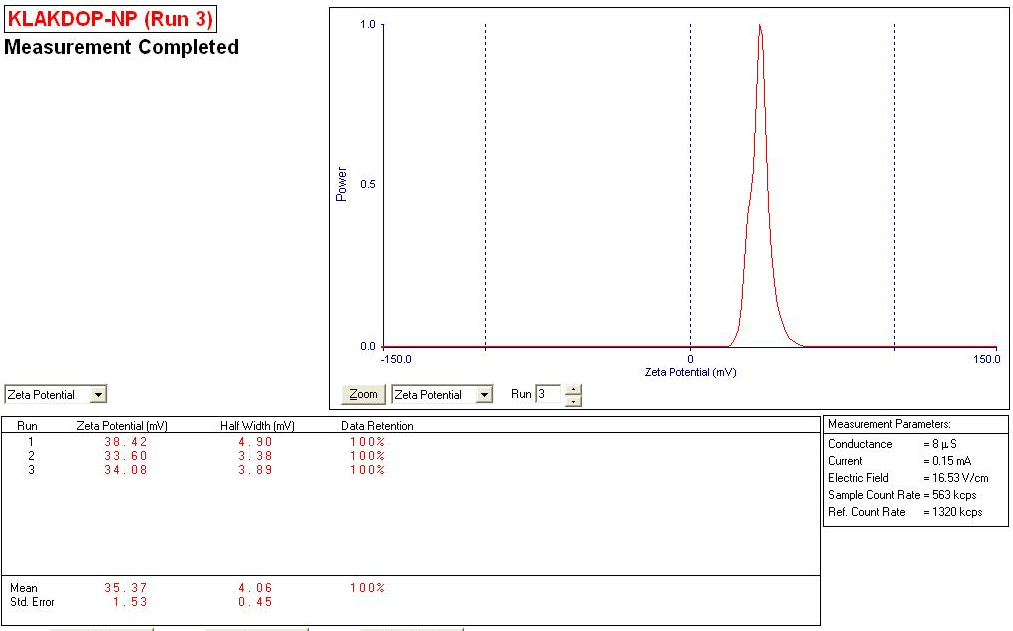
**

**Figure S11.** Zeta potential of of PLFAER_D_[KLAKLAK]_2_**C**GKRK-tethered dopamine-coated Fe/Fe_3_O_4_ in PBS (Dop-Fe/Fe_3_O_4_ Peptide), see Table 2.


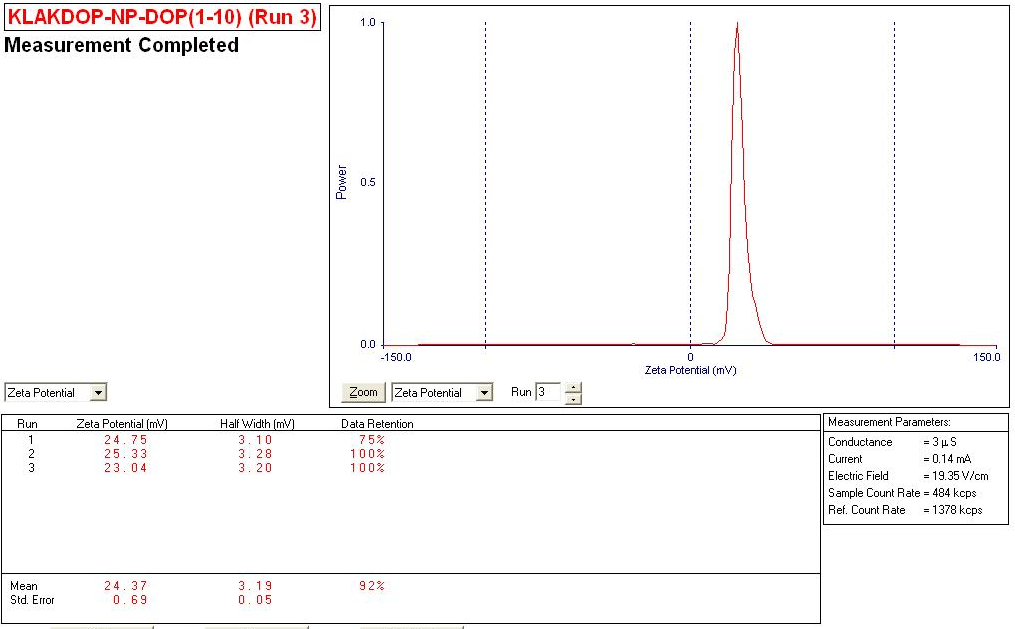


**Figure S12.** Zeta potential of PLFAER_D_[KLAKLAK]_2_**C**GKRK- and Dp44mT-derivative- tethered dopamine-coated Fe/Fe_3_O_4_ (Dop-Fe/Fe_3_O_4_ Peptide/Dp44mT (1:10)), ratio of Peptide to Dp44mT = 1/103, see Table 2.


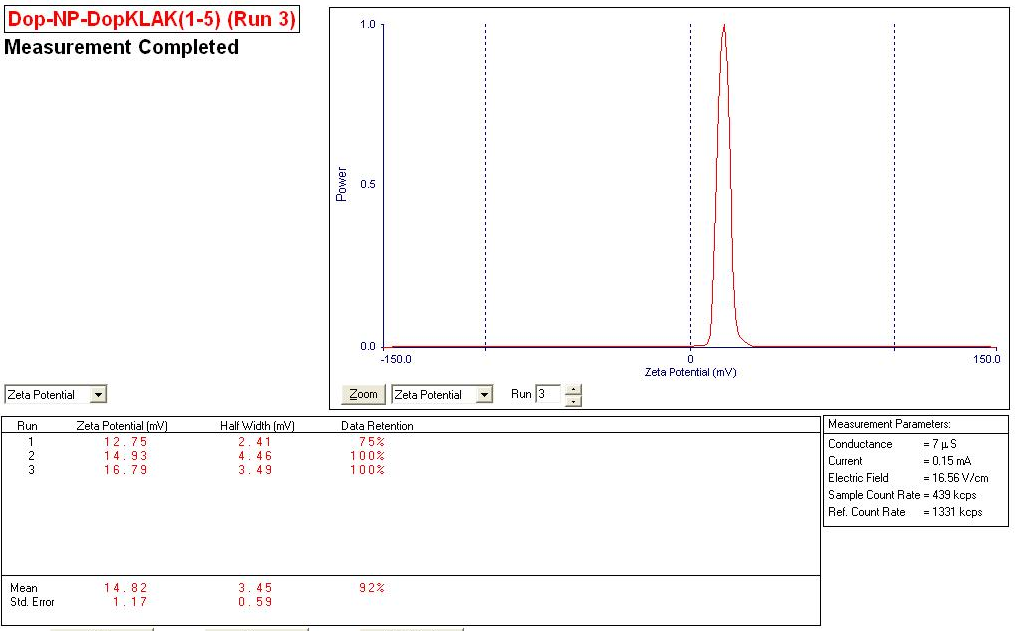


**Figure S13.** Zeta potential of PLFAER_D_[KLAKLAK]_2_**C**GKRK- and Dp44mT-derivative- tethered dopamine-coated Fe/Fe_3_O_4_ (Dop-Fe/Fe_3_O_4_ Peptide/Dp44mT (1:5)), ratio of Peptide to Dp44mT = 1/74, see Table 2.


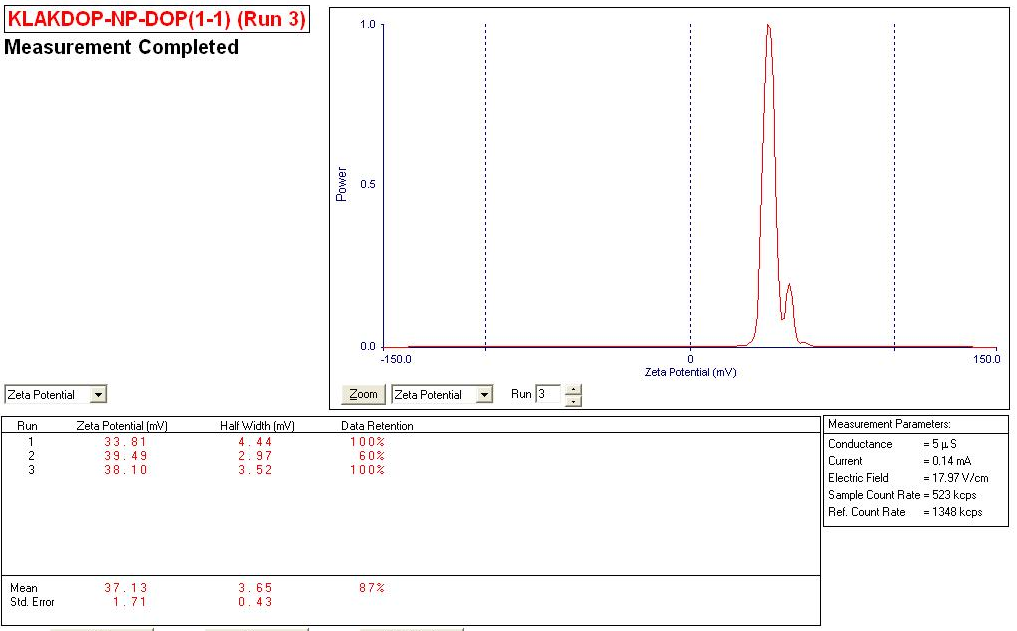


**Figure S14.** Zeta potential of PLFAER_D_[KLAKLAK]_2_**C**GKRK- and Dp44mT-derivative- tethered dopamine-coated Fe/Fe_3_O_4_ (Dop-Fe/Fe_3_O_4_ Peptide/Dp44mT (1:1)), ratio of Peptide to Dp44mT = 1/27, see Table 2.


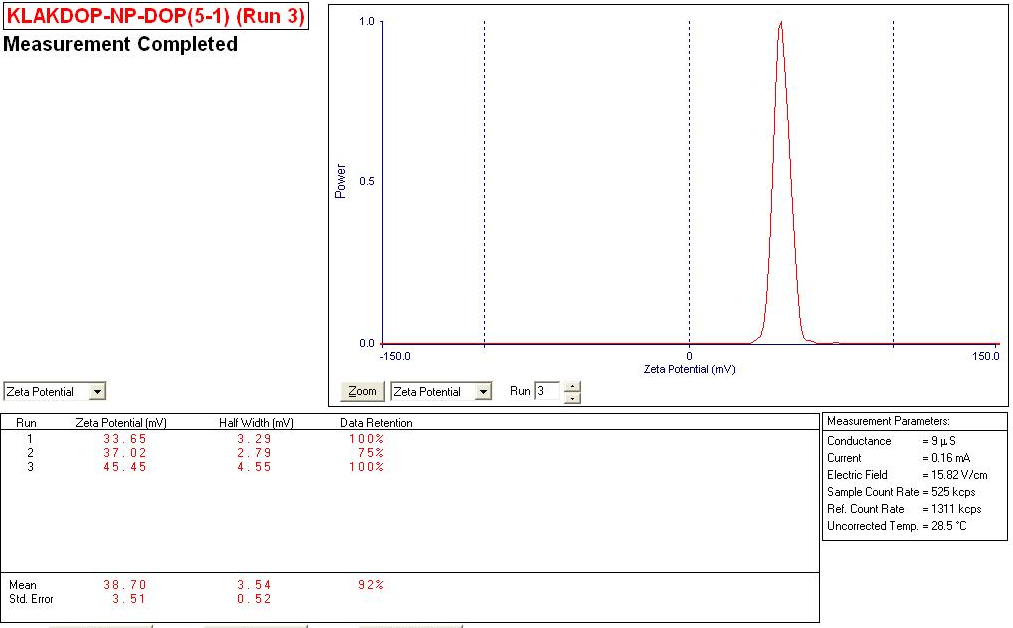


**Figure S15.** Zeta potential of PLFAER_D_[KLAKLAK]_2_**C**GKRK- and Dp44mT-derivative- tethered dopamine-coated Fe/Fe_3_O_4_ (Dop-Fe/Fe_3_O_4_ Peptide/Dp44mT (5:1)), ratio of Peptide to Dp44mT = 1/3.2, see Table 2.


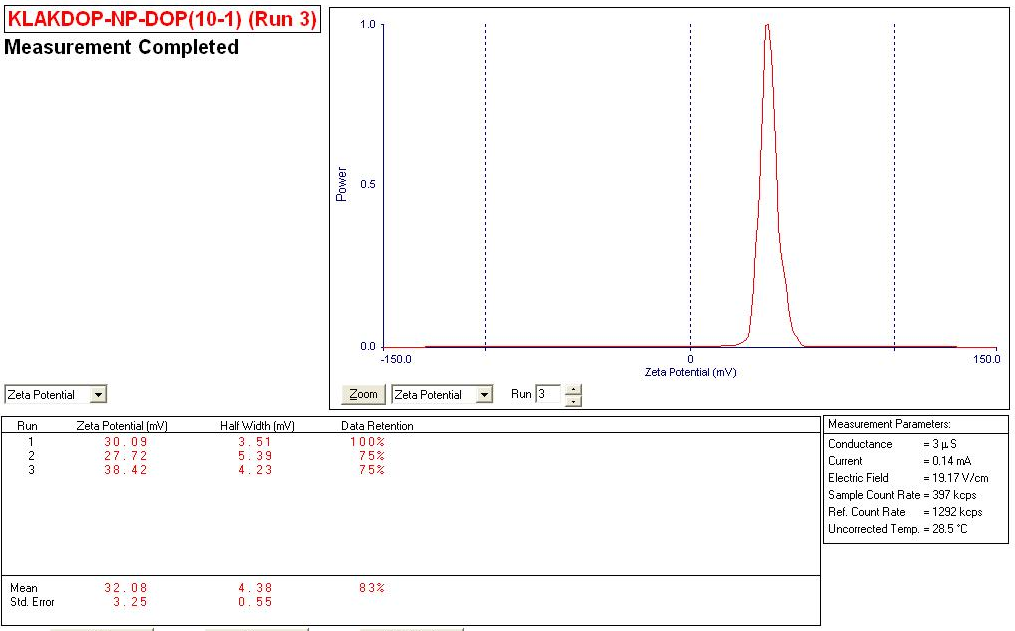


**Figure S16.** Zeta potential of PLFAER_D_[KLAKLAK]_2_**C**GKRK- and Dp44mT-derivative- tethered dopamine-coated Fe/Fe_3_O_4_ (Dop-Fe/Fe_3_O_4_ Peptide/Dp44mT (10:1)), ratio of Peptide to Dp44mT = 1/1.1, see Table 2.


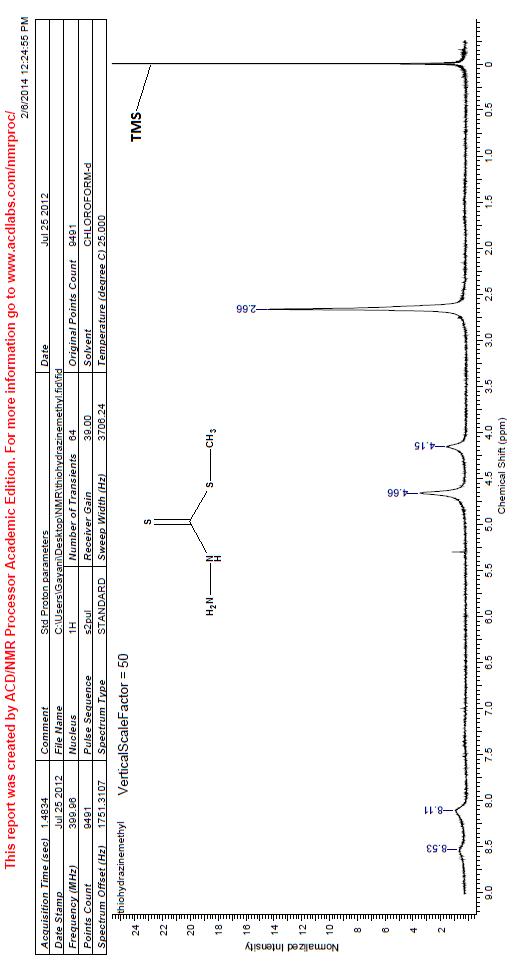


**Figure S17.** ^1^HNMR of Hydrazinecarbodithioic acid methylester ^1^H NMR (CDCl_3_-d, δ ppm) 2.66 (s, 3H, CH_3_), 4.15(s, 2H, NH_3_^+^ (protonated nitrogen) D_2_O exchangeable) 4.66 (s, 2H, NH_2_, D_2_O exchangeable), 8.11 (s, H, NH_2_^+^CS (protonated nitrogen), D_2_O exchangeable), 8.53 (s, H, NHCS, D_2_O exchangeable).


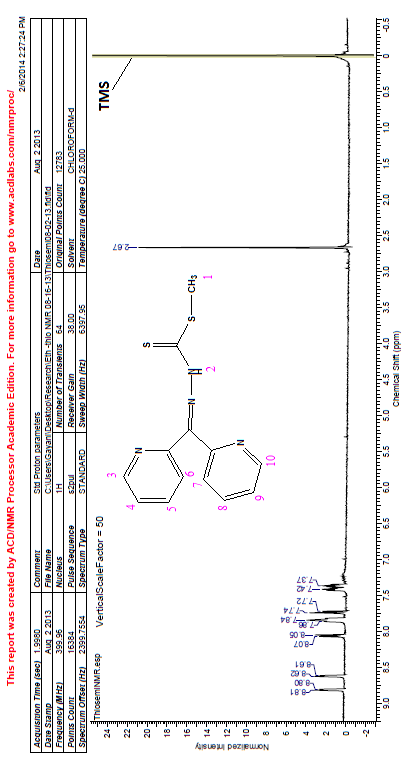


**Figure S18.** ^1^HNMR of the thiosemicarbazone iron chelator (compound **3**) N´-(Di-pyridin-2yl-methylene)- hydrazinecarbodithioic acid methylester. ^1^H NMR (CDCl3, δ ppm) 2.67 (s, 3H, CH_3_); 7.52 (s, H, NH); 8.81 (d, H3), 8.62 (d, H8), 8.06 (d, H6), 7.84 (m, H5 & 8), 7.73 (d, H7), 7.42 (m, H4), 7.37 (m, H9) of the Ar-H.


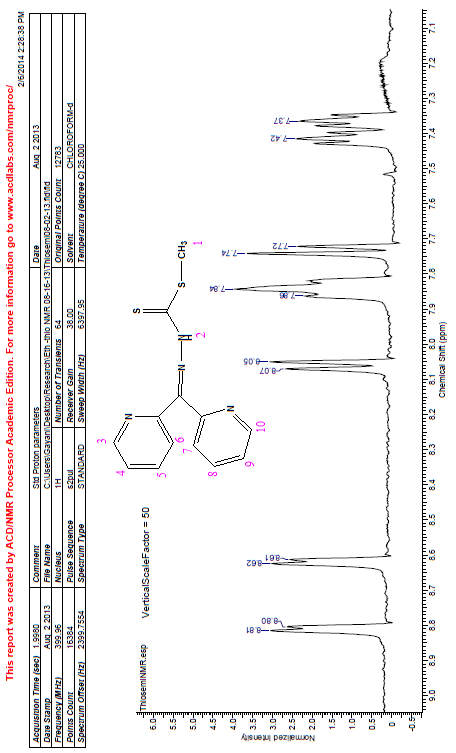


**Figure S19**. ^1^HNMR of the aryl hydrogens of the thiosemicarbazone iron chelator, N´-(Di-pyridin-2yl-methylene)- hydrazinecarbodithioic acid methylester. ^1^H NMR (CDCl3, δ ppm) 2.67 (s, 3H, CH_3_); 7.52 (s, H, NH); 8.81 (d, H3), 8.62 (d, H8), 8.06 (d, H6), 7.84 (m, H5 & 8), 7.73 (d, H7), 7.42 (m, H4), 7.37 (m, H9) of the Ar-H.


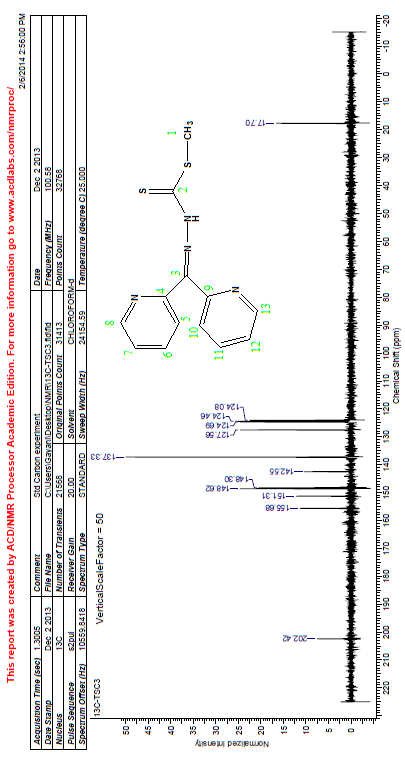


**Figure S20**. ^13^CNMR of the thiosemicarbazone iron chelator N´-(Di-pyridin-2yl-methylene)- hydrazinecarbodithioic acid methylester. ^13^C NMR (CDCl_3_, δ ppm, J, Hz): 17.70 (CH_3_); 124.08 (C10), 124.46 (C5), 124.69 (C12), 127.56 (C7), 137.33 (C6 & 11), 142.55 (C13), 148.30 (C8), 148.62 (C9), 151.31 (C4) of the Ar-C; 155.68 (C=N); 202.44 (C=S).
